# Virion proteomics of genetically intact HCMV reveals a regulator of envelope glycoprotein composition that protects against humoral immunity

**DOI:** 10.1073/pnas.2425622122

**Published:** 2025-09-18

**Authors:** Kirsten Bentley, Evelina Statkute, Isa Murrell, Ceri A. Fielding, Robin Antrobus, Hannah Preston, Lauren Kerr-Jones, Daniel Cochrane, Ilija Brizic, Paul J. Lehner, Gavin W. G. Wilkinson, Eddie C. Y. Wang, Stephen C. Graham, Michael P. Weekes, Richard J. Stanton

**Affiliations:** ^a^Division of Infection and Immunity, School of Medicine, Cardiff CF14 4XN, United Kingdom; ^b^Cambridge University Institute for Medical Research, Cambridge University, Cambridge CB2 0XY, United Kingdom; ^c^Center for Proteomics, School of Medicine, University of Rijeka, Rijeka 51000, Croatia; ^d^Cambridge Institute for Therapeutic Immunology and Infectious Disease, Cambridge University, Cambridge CB2 0AW, United Kingdom; ^e^Department of Pathology, University of Cambridge, Cambridge CB2 1QP, United Kingdom

**Keywords:** HCMV, glycoproteins, proteomics, ADCC, entry

## Abstract

Human cytomegalovirus (HCMV) is a clinically important herpesvirus that has coevolved for millions of years with its human host, and establishes lifelong persistent infection. A substantial proportion of its 235 kb genome is dedicated to manipulating host immunity through targeting antiviral host proteins for degradation or relocalization. Quantitative proteomics of the infected cell has extensively characterized these processes, but the cell-free virion has been less well studied. We therefore carried out proteomic analysis of a clinical HCMV strain (Merlin) virion. This revealed 18 novel components, including the viral protein gpUL141, which is recognized as an NK immune-evasin that targets several host proteins (CD155, CD112, and TRAILR) when expressed within the cell. Coimmunoprecipitation of gpUL141 from virions identified interactions with viral entry glycoproteins from the trimer (gH/gL/gO), pentamer (gH/gL/UL128/UL130/UL131A), and gH/gpUL116 complexes, as well as gB. Only interactions with gH/gB occurred in the absence of other viral proteins. Analysis supported a model in which gpUL141 homodimers independently interacted with separate gB/gH-containing complexes. gpUL141 encodes an ER retention domain that restricts trafficking through the ER/Golgi, and limited the transport of glycoprotein complexes bound by gpUL141. As a result, gpUL141 reduced levels of multiple glycoprotein complexes on the infected cell surface as well as in the virion. This reduced syncytium formation, inhibited antibody-dependent cellular cytotoxicity (ADCC), and reduced susceptibility to neutralizing antibodies. Thus, gpUL141 represents an immune-evasin that not only targets host proteins to limit NK-cell attack, but also alters the trafficking of multiple viral glycoprotein complexes in order to evade humoral immunity.

## Significance

The large number of immune-modulators encoded by HCMV has led to it becoming a paradigm for pathogen-mediated immune-modulation. These mechanisms have informed on virus pathogenesis, the evolution and function of host defenses, and identified therapeutic targets. Previously discovered immune-evasins functioned by modifying host proteins. gpUL141 represents a novel strategy in which multiple immunological functions are targeted through manipulating eight different viral entry glycoproteins, via interactions with two proteins common to multiple complexes. Entry glycoproteins define virus tropism, mechanisms of infection and spread, and susceptibility to neutralizing and nonneutralizing antibody activities, yet UL141 is frequently mutated in passaged viruses. This demonstrates the need to work with virus strains encoding the complete repertoire of viral accessory proteins during preclinical therapeutic development.

Human cytomegalovirus (HCMV) is a clinically significant betaherpesvirus that establishes lifelong persistence in the majority of people worldwide. It is a significant cause of morbidity and mortality in the immunocompromised, and the leading infectious cause of congenital malformation. To date, no vaccine is licensed, and antivirals are limited by toxicity and the selection of resistance mutations.

HCMV has the largest genome of any human virus and has coevolved with its human host over millions of years. This has enabled it to develop an exceptionally broad range of mechanisms to manipulate host immunity to promote persistence. Only 41 to 45 of the 170 canonical ORFs are essential for replication in vitro, with the remainder providing accessory functions that are required to promote persistence in vivo (1, 2). These include proteins that prevent cell death, inhibit innate and intrinsic immunity, limit T cell recognition, and prevent NK-cell attack, through targeting host antiviral proteins for degradation or relocalization (3–5). As a result of these activities, HCMV has become a paradigm for pathogen-mediated immune-evasion.

A challenge of studying these viral accessory functions in HCMV is that mutations in nonessential genes are invariably selected when clinical viruses are passaged in vitro. Although the loss of a 15 kb genetic region encoding >20 genes in the AD169 strain is perhaps the most extreme example, even short-term passage rapidly selects for mutations (6). As a result, laboratory strains differ both genotypically and phenotypically from the causative agent of disease (7). To address this problem, we previously generated an infectious bacterial artificial chromosome (BAC) clone of a HCMV strain (Merlin) for which we had the sequence of the original clinical material (7, 8). This allowed us to correct in vitro acquired mutations and regenerate virus containing a genome that exactly matched the patient sample. Like clinical isolates, this virus also accrued mutations upon passage in vitro, with RL13 and a complex of three genes, UL128, UL130, UL131A (the UL128 locus; UL128L), the most rapidly selected. Engineering of the Merlin BAC enabled us to selectively repress these genes when virus stocks were grown in vitro, permitting recovery of high titer virus for experimental use without risk of mutation.

Study of strain Merlin has led to the discovery of multiple phenomena which differ between passaged and clinical viruses, including extensive virus-induced modulation of the cell-surface proteome through metalloproteinase manipulation (9), novel cell-surface therapeutic targets (10), immune-evasion mediated by actin manipulation (11), functions that prevent natural killer cell attack (12–14), and mechanisms that promote a form of cell-associated spread that is highly resistant to neutralizing antibodies (8, 15). Many of these discoveries were underpinned by extensive use of quantitative proteomics analysis to understand how HCMV manipulates the infected cell to drive viral survival following entry (16–27). However, the virion itself has not been subject to the same level of investigation. Previous mass spectrometry analyses have been carried out using the passaged AD169 (28, 29) and TB40 (30, 31) strains. However, given the existence of mutations in these strains, we carried out an analysis of virions derived from the Merlin BAC with the intention of producing a wildtype HCMV virion proteome. We unexpectedly found that glycoprotein UL141 (gpUL141) is a novel virion component. Although previously demonstrated to manipulate host ligands (CD155, CD112, and TRAILR) to limit NK activation (12–14), we now show that it has also been co-opted by HCMV to regulate the trafficking of the majority of viral entry glycoproteins to the cell surface and the virion particle in order to provide resistance to multiple different aspects of humoral immunity.

## Results

### The Wildtype HCMV Virion Contains Multiple Proteins Absent From Passaged Strains.

**Fig. 1.**
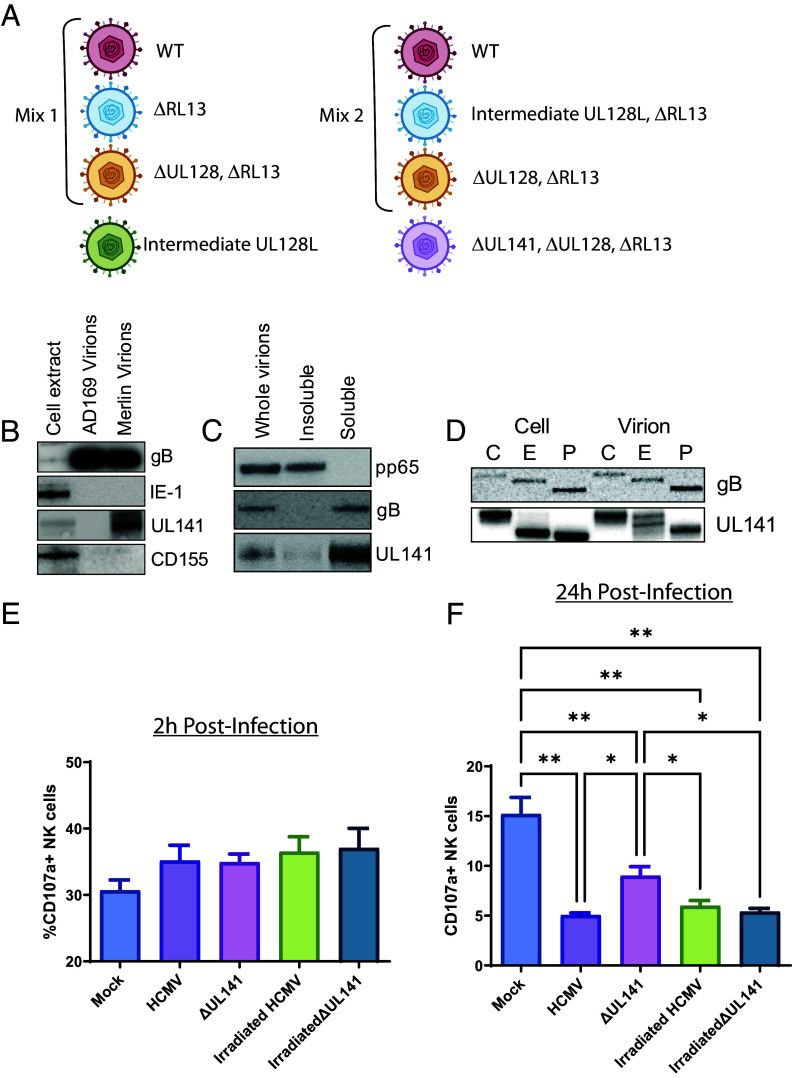
gpUL141’s presence in the virion envelope does not lead to NK inhibition. (*A*) Schematic of virions analyzed in this study. (*B*) Virions from the passaged AD169 strain, or the wildtype Merlin strain, were purified from HFFF and analyzed by western blot. (*C*) Virions from strain Merlin were purified from HFFF then treated with detergent to extract membrane proteins, before assessing by western blot. (*D*) Virions from strain Merlin were purified from HFFF then left untreated (*E*) or digested with EndoH (E) or PNGaseF (P) before analyzing by western blot. (*E* and *F*) cell-free strain Merlin virions were added to HFFF for 2 h (*E*) or 24 h (*F*), then cells were coincubated with NK cells for 5 h in the presence of CD107a antibody and golgistop, before levels of NK-cell degranulation were assessed.

To define the complete repertoire of proteins associated with the strain Merlin virion, we used mass spectrometry to analyze virions that had been separated from noninfectious particles and dense bodies on glycerol-tartrate gradients. We individually analyzed six Merlin variants that we were working with as part of previous studies: a wildtype genome, a genome mutated for RL13, and a genome mutated for both RL13 and UL128L, to assess alterations deriving from the presence or absence of the pentameric complex and/or gpRL13 (8); two viruses were included that contained point mutations in an intron of UL128 that result in intermediate UL128L expression levels enabling comparisons with passaged strains of HCMV that retain pentamer expression but at lower levels (32); and a virus lacking UL141 (13). In addition to analyzing virion preparations individually, quantitative comparisons were made by producing virions in media containing different isotopes of arginine and lysine (Stable Isotope Labelling of Amino Acids in Culture; SILAC), then analyzing as two groups of three variants (Fig. 1*A* and Dataset S1).

These SILAC analyses of near identical Merlin virions, differing only in RL13 and UL128L, indicated that deletion of RL13 made very little difference to the relative abundance of virion proteins while loss of UL128L led to higher levels of gO (as previously reported) (32, 33) and UL146 (*SI Appendix*, Fig. S1). Aside from this, there were no consistent differences that would support effects of these proteins on other virion components. The absolute Merlin virion proteome was determined as the consensus of proteins identified across all virion preparations. 96 viral proteins were designated as “high confidence” when the peptides were present in at least half of all virus preparations (*SI Appendix*, Table S1). Of these, 53 to 68 were previously identified by mass spectrometry analyses of AD169 virions (28, 29) and 35 by mass spectrometry analysis of TB40 (30, 31). A further four proteins not identified by mass spectrometry analysis of AD169 or TB40 virions, but recognized as HCMV virion components following assessment of virions by alternative approaches, were also detected with high confidence [UL36, gpRL13 (8), gpUL130 (34, 35), UL23 (36)]. 18 proteins identified with high confidence have not been previously described as virion components, with at least 10 membrane-localized. Some of these have been found as virion components in CMVs from other species, however others are unique to HCMV (*SI Appendix*, Table S1).

We used iBAQ (intensity-based absolute quantitation) values for virions with a wild-type genome to estimate the relative abundance of different virion components. Certain proteins accounted for a substantial overall proportion of the total protein content of the virion. These included tegument proteins pp65 (~43%), pp71 (~4%), and pp150 (~1.5%) as well as capsid proteins UL86 (~5%) and UL85 (~4%). To determine which viral components were specifically enriched in the virion in contrast to lysates of infected cells, we compared iBAQ values for virion proteins with those we previously calculated for cells infected with Merlin-strain HCMV (22). Most proteins exhibited similar abundance, but there was a 2-340 fold enrichment of multiple virion membrane proteins, as well as capsid and tegument proteins. These included membrane proteins gN, gM, gH, and gL, capsid proteins UL46, UL47, and UL85 as well as tegument proteins UL83, UL82, and UL103 (*SI Appendix*, Fig. S2 *A* and *B* and Dataset S2*A*). There was also enrichment of certain types of cellular proteins, particularly those with transmembrane domains, lipoproteins, ATP synthase subunits and host receptors for viral entry. The latter included known receptors for HCMV including Integrins β1 and α2 (*SI Appendix*, Fig. S3 *A* and *B* and Dataset S2 *B* and *C*).

### gpUL141 Is a Novel Virion Envelope Glycoprotein, but Does Not Act as an Immune-Evasin When Delivered by the Virion.

We have previously characterized the viral protein gpUL141 as a potent inhibitor of NK activation. When expressed within the cell, it binds CD155 and all four TRAIL receptors and prevents surface expression by retaining them within the ER (12, 14). This has the effect of rendering cells more resistant to TRAIL-mediated apoptosis and NK-cell attack. It also co-operates with US2 to target CD112 for degradation, preventing NK-cell activation (13, 17). We were therefore interested to note that our proteomics analysis identified gpUL141 as a novel virion component. It may not have previously been identified as such because many HCMV strains have acquired mutations during in vitro passage that abrogate UL141 expression (6, 37). Western blot analysis confirmed the presence of gpUL141 in purified virions in the absence of its cellular ligand CD155 (Fig. 1*B*). Fractionation of virions confirmed that it was in the envelope compartment (Fig. 1*C*), consistent with the fact that it encodes a transmembrane domain and a signal peptide. Interestingly, gpUL141 found in the virion was resistant to EndoH, while cell-associated gpUL141 was sensitive (Fig. 1*D*), suggesting that gpUL141 is present as two fractions—one is retained within the ER within the cell, while the other must traffic through the ER and the Golgi to be incorporated into the virion.

Given the characterized role of gpUL141 as an immune-evasin, we investigated whether virion-delivered gpUL141 could act as a rapid immune-evasin, prior to de novo gene expression. Cells were infected with wildtype HCMV or HCMV lacking UL141, either as live or irradiated virus. Cells were then cocultured with NK cells and NK-degranulation assessed. NK activation was not affected by the presence or absence of UL141, nor the ability of the virus to initiate de novo gene expression, at 2 h postinfection (Fig. 1*E*). At 24 h postinfection, loss of UL141 led to increased NK activation, but this was not seen in the irradiated virus (Fig. 1*F*). Thus, this effect was due to gpUL141 synthesized de novo within the infected cell. We therefore concluded that virion delivered gpUL141 does not impact NK cell activity, only gpUL141 expressed de novo within the cell exhibited this property.

### gpUL141 Interacts With Multiple HCMV Entry Glycoproteins Within the Virion.

**Fig. 2.**
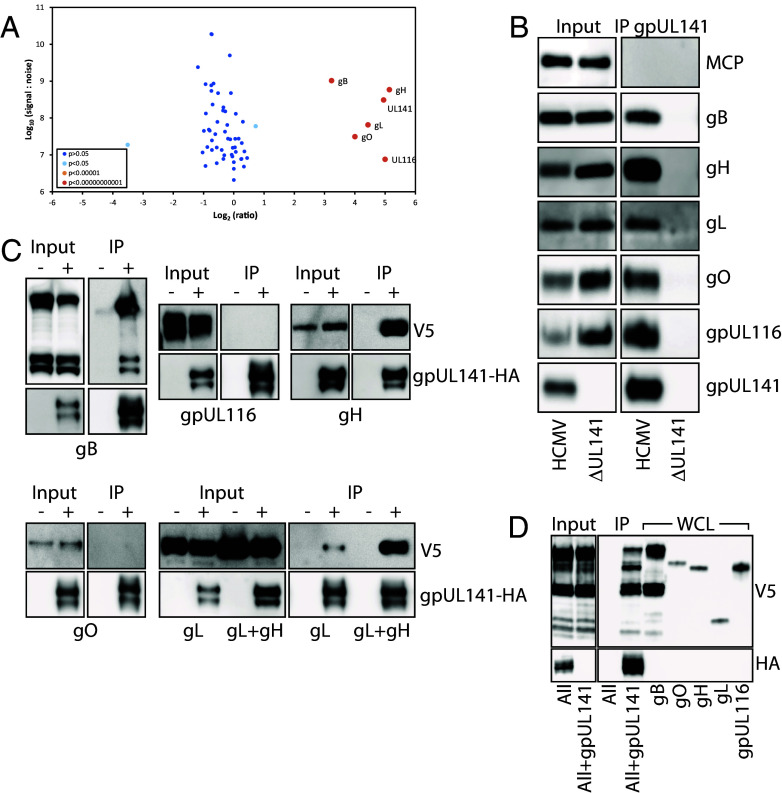
gpUL141 interacts with multiple entry glycoprotein complexes. (*A*) Cell-free strain Merlin virions that lacked UL128L but contained either a C-terminal V5 tag on gpUL141, or a deletion of UL141, were grown in HFFF in “Medium” or “Heavy” SILAC Media respectively, then purified on glycerol-tartrate gradients and proteins subjected to IP with a V5 antibody. Samples were then analyzed by MS, and the ratio of identified proteins between the two samples calculated. (*B*) Virions were prepared in the same way as (*A*) but without SILAC media, then analyzed by western blot. (*C* and *D*) RAd vectors expressing all potential gpUL141 interactors each with a C-terminal V5 tag, or gpUL141 with a C-terminal HA tag, were used to coinfect HFFF. (*C*) Each potential interactor was expressed alone (−) or with (+) gpUL141, then gpUL141 IP’d using an anti-HA antibody, before assessing by western blot. (*D*) All interacting proteins were expressed together from RAds in the absence or presence of gpUL141, then IP performed for gpUL141, followed by western blot analysis. To identify individual proteins in the mix based on size, whole-cell-lysates (WCL) of each protein expressed alone are provided alongside.

To investigate what roles virion-associated gpUL141 might play, we carried out a SILAC-immunoprecipitation (SILAC-IP) in which gpUL141 was pulled down via a C-terminal V5 tag from purified virions, then analyzed by MS to identify interacting proteins. A virus lacking pentamer expression was used due to the higher cell-free titers it produces, with analysis revealing interactions of gpUL141 with gB, gH, gL, gO, and gpUL116 (Fig. 2*A* and Dataset S3). gB is the conserved herpesvirus fusogen, gH/gL/gO form the “Trimer” complex which is essential for cell-free entry into all cell types via interactions with PDGFRa as well as other (unidentified) receptors (38, 39), while gpUL116 interacts with gH but has no known receptor binding role currently (40–42). All interactions were confirmed by IP-western of gpUL141 from purified virions (Fig. 2*B*). Thus, gpUL141 interacts with multiple members of the HCMV entry machinery within the virion.

To determine which interactions with gpUL141 might be direct, gpUL141 was expressed from an adenovirus vector along with each protein from the SILAC-IP individually, then immuno-precipitated. Only gH and gB were robustly coimmunoprecipitated with UL141 in this pairwise test, indicating a potentially direct interaction. Although some interaction was observed with gL, this was significantly weaker than when gH and gL were coexpressed, indicating only a weak interaction. All other proteins are presumably pulled down by indirect interactions via gH/gB (Fig. 2*C*). Consistent with this, when all proteins were expressed together from an adenovirus vector, all coimmunoprecipitated with gpUL141, indicating that no other viral proteins are required for all complexes to form (Fig. 2*D*).

**Fig. 3.**
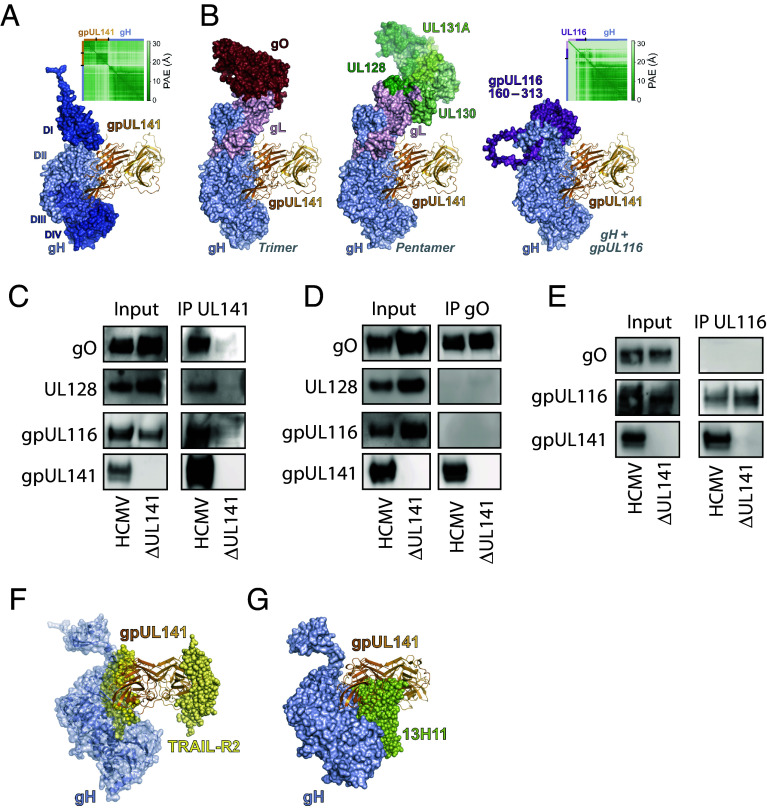
gpUL141 interacts independently with all major gH-containing glycoprotein complexes. (*A*) Predicted structure of gH (blue surface, colored by domain) in complex with a homodimer of gpUL141 (orange ribbons). Inset shows the predicted aligned error (PAE) of the model. (*B*) Superposition of gpUL141 from the predicted gH:gpUL141 structure onto the electron cryomicroscopy structure of the trimer [PDB 7LBE (39), *Left*], the crystal structure of the pentamer [PDB 5VOB (47), *Middle*], and a predicted complex of gH with UL116 (*Right*). For clarity, the N-terminal region of UL116 that is predicted to be highly unstructured is not shown. Inset shows the PAE of the gH:UL116 model. (*C*) HFFF were infected with strain Merlin containing either a C-terminal V5 tag on UL141, or a deletion of UL141, for 96 h then proteins subjected to IP with an anti-V5 antibody followed by western blot. (*D*) HFFF were infected with strain Merlin containing or lacking UL141 expression for 96 h, then proteins subjected to IP with an anti-gO antibody followed by western blot. (*E*) HFFF were infected with strain Merlin expressing a C-terminal HA tag on gpUL116 and either containing or lacking gpUL141 expression for 96 h, then proteins subjected to IP with an anti-HA antibody followed by western blot. (*F*) Superposition of the predicted gH (blue semitransparent surface and ribbons) plus gpUL141 (orange ribbons) complex onto the crystal structure of gpUL141 in complex with the death receptor TRAIL-R2 [yellow spheres; PDB 4I9X (12)]. (*G*) Superposition of gpUL141 from the predicted gH:gpUL141 structure (orange ribbons) onto the electron cryomicroscopy structure of gH (blue surface) bound to the Fab region of antibody 13H11 [lime green spheres; PDB 7LBE (39)].

Deep learning structure prediction using AlphaFold3 (AF3) (43) was used to further understand the potential direct physical interactions between gpUL141 and gH or gB. Previous structural characterization of gpUL141 has indicated that it forms a head-to-tail homodimer when in complex with its cellular targets CD155 or TRAILR2 (44, 45), hence two copies of the gpUL141 sequence were included in all structure predictions. AF3 predicted a high confidence interaction between gH and the gpUL141 dimer, with gpUL141 contacting domains DI, DII, and DIII of gH (Fig. 3*A* and *SI Appendix*, Fig. S4). This prediction is supported by a recently published experimental structure which shows a high level of overlap (*SI Appendix*, Fig. S4) (46). AF3 did not predict a high confidence interaction between gpUL141 and gB (*SI Appendix*, Fig. S4), potentially because AF3 predictions yielded a postfusion conformation of gB rather than the prefusion complex likely to predominate in HCMV virions.

### gpUL141 Interacts Independently With All Major gH-Containing Glycoprotein Complexes.

HCMV encodes three gH-containing complexes—trimer (gH/gL/gO), pentamer (gH/gL/UL128/UL130/UL131A), and UL116 (gH/UL116)—which are mutually exclusive; UL128 and gO compete for the same binding sites on gL (48), while gL competes with gpUL116 for binding to gH (40). The AF3-predicted binding of gpUL141 would not be expected to inhibit the interaction between gH and either gL or gpUL116 (Fig. 3*B* and *SI Appendix*, Fig. S4), consistent with our immunoprecipitation experiments, implying that gpUL141 interacts with both gpUL116 and the trimer via gH. This structural model would also imply that gpUL141 could in addition interact with pentamer via gH (Fig. 3*B*). Indeed, when gpUL141 was immunoprecipitated from infected cells, it pulled down gpUL116, gO, and UL128, indicative of interactions with all recognized gH-containing complexes (Fig. 3*C*).

The predicted gpUL141:gH structure is consistent with dimers of gpUL141 symmetrically binding two gH molecules. In this case it could potentially “bridge” different gH complexes (*SI Appendix*, Fig. S4). To determine whether this occurs we immunoprecipitated gO, then stained for gpUL116 or UL128. Whereas immunoprecipitation of UL141 pulls down all of these proteins (Fig. 2 *B* and *C*), pulling down gO did not pull down gpUL116 or UL128 (Fig. 3*D*). Similarly, pulling down gpUL116 did not coprecipitate gO (Fig. 3*E*). Therefore, gpUL141 interacts with all three gH containing complexes (Trimer, Pentamer, and gpUL116), but does not link different complexes together. In contrast, the predicted gH binding interface of gpUL141 overlaps significantly with the surface bound by TRAIL-R2 (Fig. 3*F*), suggesting that binding of gpUL141 to gH (and complexes thereof) and TRAIL death receptors would be mutually exclusive, consistent with virion SILAC-IP data (Fig. 2*A*) and western blot (Fig. 1*B*). Interestingly, the predicted gH/UL141 interface also overlaps with the binding site of a gH neutralizing antibody [13H11 (49)], indicating that UL141 may impact on epitope availability for antiviral antibodies (Fig. 3*G*).

### gpUL141 Modulates Cell-Surface and Virion gH-Complex Levels.

**Fig. 4.**
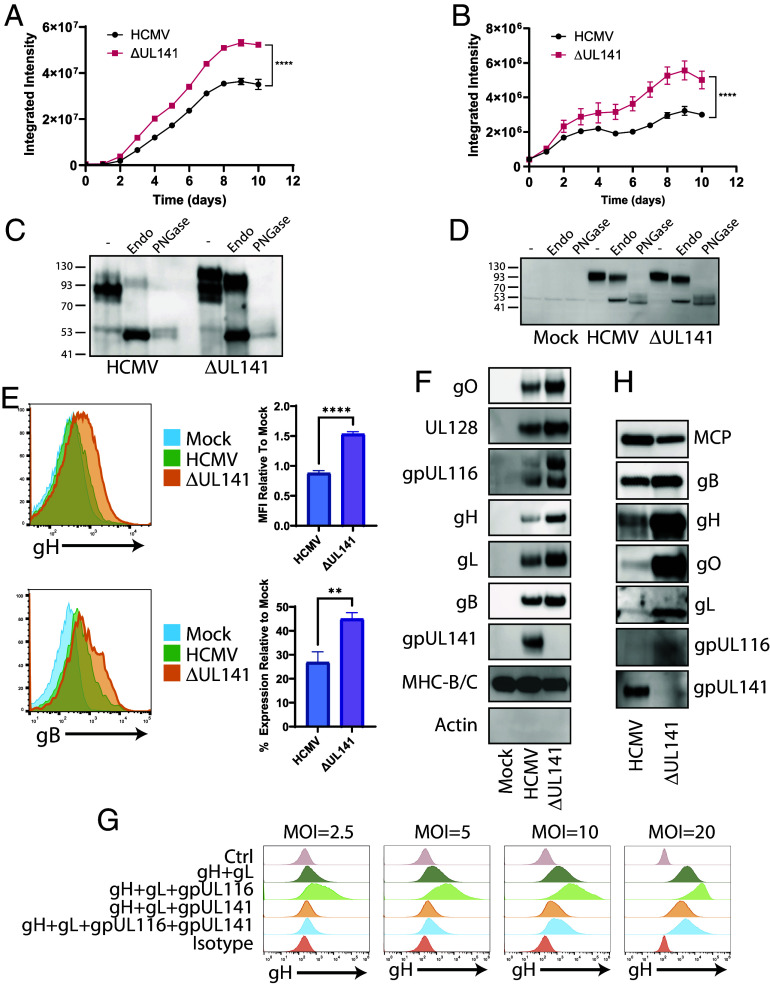
gpUL141 alters the glycoprotein composition of both the virion and the infected cell-surface. (*A* and *B*) HFFF were infected with strain Merlin-GFP with UL141 either intact or deleted, at high MOI. 72 h later cells were cocultured with RPE-1 (*A*) or HUVEC (*B*). Once the HFFF had lysed (192 h), virus dissemination through the RPE-1 or HUVEC monolayer was assessed by Incucyte. (*C*) RPE-1 cells were infected as in (*A*), 192 h post-coculture infected RPE-1 cells were subjected to digestion with EndoH or PNGaseF, or left under control conditions, then analyzed for gO protein by western blot. (*D*) HFFF were infected at high MOI with strain Merlin containing or lacking UL141, then 96 h later subjected to digestion with EndoH or PNGaseF, or left under control conditions, and analyzed for gO protein by western blot. (*E*) HFFF were infected for 72 h with strain Merlin containing or lacking gpUL141, then levels of gH and gB on the cell surface assessed by flow cytometry. (*F*) HFFF were infected for 72 h at high MOI with strain Merlin containing or lacking UL141, then cell surface proteins isolated by Plasma-membrane profiling (PMP) followed by western blot. (*G*) HFFF-CAR were infected with RAd vectors expressing the indicated proteins for 48 h, then stained for cell surface gH levels and assessed by flow cytometry. In all cases RAd-UL141 was used at MOI = 20, all other RAds were used at the indicated MOI. (*H*) HFFF were infected for 72 h at high MOI with strain Merlin either expressing or lacking gpUL141, then cocultured with uninfected RPE-1 cells. Virus was allowed to disseminate through the RPE-1 monolayer until 100% CPE was achieved, at which point virions were purified and analyzed by western blot.

We have previously shown that knocking UL141 out of the virus genome has no apparent impact on cell-free virus levels in fibroblasts, but enhances titers in epithelial cells (37). This phenomenon was not restricted to epithelial cells (Fig. 4*A*), virus dissemination was also enhanced in the absence of UL141 in endothelial cells (Fig. 4*B*), suggesting that it occurs in all cell-types where entry is dependent on pentamer. Since gpUL141 can inhibit virus dissemination and interacts with multiple gH-containing complexes that are required for virus infection, we investigated whether these phenomena were linked. The majority of gpUL141 is retained within the ER where it prevents transit of CD155 and TRAILR2 through the ER-Golgi apparatus (12, 14), yet our data also indicated that virion envelope glycoproteins must traffic through the ER and Golgi in order to become incorporated in the virion envelope (Fig. 4*C*). We therefore investigated whether the gpUL141 retained in the ER also bound gH-containing complexes and affected their transition through the ER/Golgi. We took advantage of the fact that gO is highly glycosylated, and therefore trafficking from the ER through to the Golgi can be assessed by measuring sensitivity to EndoH. When epithelial cells were infected with HCMV lacking UL141 there was a significant fraction of EndoH resistant gO, which was reduced in virus expressing UL141, indicating that more gO is retained in the ER in the presence of gpUL141 (Fig. 4*C*).

Although we had investigated this phenomenon in epithelial cells due to the restriction in virus dissemination observed in this cell type, there was no reason to assume that it was restricted to epithelial cells. When a comparable assay was carried out in fibroblasts, we observed a similar pattern, albeit with a smaller reduction (Fig. 4*D*). We therefore expanded this result by measuring cell-surface levels of gH and gB by flow cytometry in fibroblasts. At 72 h postinfection in the presence of gpUL141, gH, and gB levels were low (gB) or undetectable (gH). However, when UL141 was deleted, surface levels of gH and gB were enhanced (Fig. 4*E*). Antibodies are not available to detect the cell-surface forms of other gH-containing complexes. To determine whether this phenomenon extended to all proteins that interact either directly or indirectly with gpUL141, we therefore used our previously validated “Plasma Membrane Profiling” technique to isolate cell-surface proteins and assess levels by western blot (16, 50). In support of EndoH and flow analysis, levels of gO, gH, gL, gpUL116, gB, and UL128 on the plasma membrane were all reduced in the presence of gpUL141, while a control protein (free heavy chains of HLA-B/C) was unaffected (Fig. 4*F*). This phenomenon could also be recapitulated outside of the context of infection, using adenovirus vectors expressing individual glycoproteins (Fig. 4*G*). When gH is expressed in isolation it does not traffic to the plasma membrane, but it becomes surface-localized when coexpressed with gL, and this is enhanced when gpUL116 is additionally coexpressed. However, in all cases, coexpression of UL141 resulted in a reduction in cell-surface levels. This was dose-dependent, with an excess of UL141 leading to almost complete inhibition of surface trafficking.

Since cell-surface trafficking was altered in the presence of gpUL141, we wondered whether this would also affect the levels of gH-containing complexes found in purified virions. Indeed, in the input samples used for our virion IP-western (Fig. 2*B*), the levels of proteins from all complexes were all significantly lower in the presence of gpUL141, with the potential exception of gB. Since the effects of gpUL141 on virus growth were more extensive in epithelial cells, we also tested a subset of proteins in virus derived from this cell type. As in fibroblasts, the levels of proteins that interacted directly or indirectly with gpUL141 were reduced within the virion (Fig. 4*H*). Thus, gpUL141 acts to reduce the incorporation of all gH containing complexes into virions, and limits presentation of these complexes (along with gB), on the plasma membrane of the infected cell.

### gpUL141-Mediated Modulation of gH-Complex Levels Is Due to Its ER Retention Domain.

**Fig. 5.**
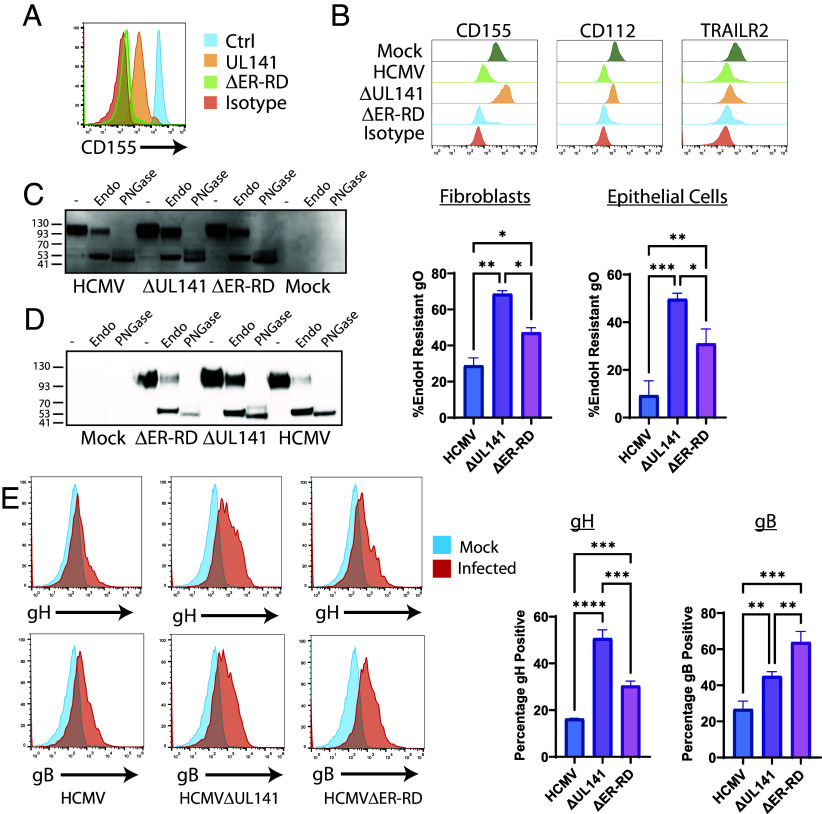
The gpUL141 ER retention domain alters envelope glycoprotein trafficking. (*A*) HFFF-CAR were infected with RAd vectors expressing wildtype UL141 or UL141 in which the C-terminal intracellular portion [containing an ER-retention domain (ER-RD)] had been deleted, then 48 h later were stained for cell-surface CD155 levels and analyzed by flow cytometry. (*B*–*E*) Cells were infected with wildtype HCMV strain Merlin or virus lacking either the whole of gpUL141, or just the C-terminal intracellular portion of gpUL141 (ER-RD). (*A*) HFFF were infected with RAd vectors encoding UL141 or ΔER-RD for 48 h or (*B*) with HCMV encoding UL141, deleted for UL141, or ΔER-RD, for 72 h and then stained with anti-CD155 (*A*) or anti-CD155, anti-CD112, or TRAILR2 antibodies (*B*) and analyzed by flow cytometry. (*C*) HFFF were infected for 96 h or (*D*) HFFF were infected for 72 h then cocultured with uninfected RPE-1 cells for a further 8d, then samples subjected to digestion with EndoH or PNGase F, or left undigested before being analyzed by western blot and stained for gO. (*E*) HFFF were infected for 96 h then stained for cell surface gH or gB followed by flow cytometry.

UL141 is retained within the ER where it prevents ER-exit of CD155 and TRAILR (12, 14). It has previously been assumed that this ER-retention function is necessary to prevent cell-surface trafficking of these cellular proteins in order to prevent activation of NK cells. However, our data imply that the ER retention function may have additional impacts on multiple viral entry glycoproteins. For transmembrane proteins, the ER retention domain is often in the C-terminus of the protein, with the classical sequence being KKXX. UL141 contains such a motif at its extreme C-terminus. To explore this further, we therefore generated HCMV viruses in which the C-terminal intracellular portion of UL141 was deleted. We have previously shown that this results in increased trafficking of gpUL141 through the ER/Golgi when expressed from a RAd (10), however surprisingly it did not inhibit the ability of RAd expressed gpUL141 to reduce cell surface levels of CD155, with levels actually down-regulated to a greater extent when the ER retention domain was deleted (Fig. 5*A*). Similarly, when we engineered this mutation into HCMV, it did not alter the ability of gpUL141 to limit cell-surface levels of CD155, TRAILR2, or CD112 (Fig. 5*B*). Therefore, the ability of UL141 to act as a NK-evasin does not require UL141 to be ER-resident.

To determine whether the ER retention domain affected gH-complex trafficking, we repeated the EndoH analysis of gO. In both fibroblast (Fig. 5*C*) and epithelial cells (Fig. 5*D*), loss of the ER retention domain led to an increase in levels of EndoH resistant gO, although not always to the level seen following complete UL141 deletion. When we assessed cell-surface trafficking of gH and gB, removal of the ER retention domain led to recovery of both proteins on the plasma membrane (Fig. 5*E*).

### Intracellular Retention of Complexes by gpUL141 Limits Syncytium Formation.

**Fig. 6.**
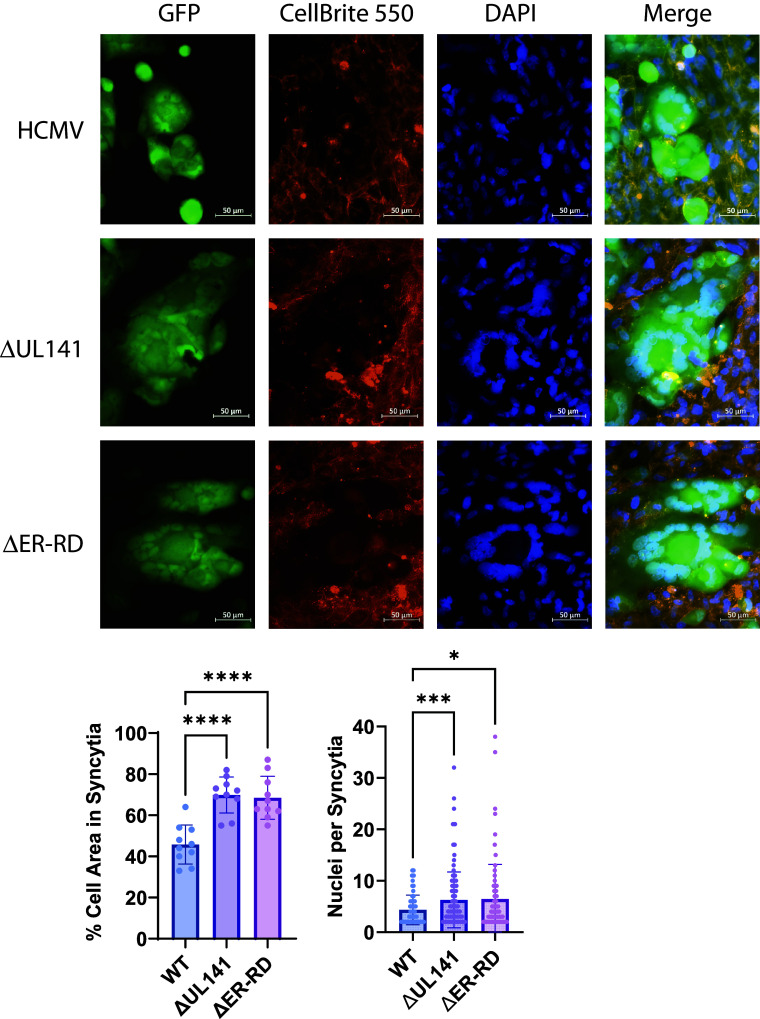
gpUL141 retention of gH complexes limits syncytia formation. HFFF were infected for 72 h at high MOI by HCMV strain Merlin expressing GFP and either containing or lacking gpUL141 expression, or expressing an ER retention domain mutant of UL141 (ΔER-RD), then cocultured with uninfected ARPE19 cells at a ratio of 0.03:1. After a further 8 d nuclei of live cells were stained with Hoechst and CellBrite® 550 Membrane stain, imaged by microscopy, and the number of syncytia quantified.

Viral entry glycoproteins on the infected cell surface can engage with entry receptors on surrounding cells leading to fusion and syncytium formation. In the case of HCMV, this requires gH/gL complexes and gB (51, 52). We reasoned that if all of these were reduced on the infected cell surface, this could reduce receptor engagement and therefore the chances of syncytium formation. ARPE-19 epithelial cells have previously been reported to readily form syncytia upon HCMV infection (52). These cells were therefore infected with GFP-expressing HCMV, and virus allowed to disseminate. Syncytia were first observed by day 6 postinfection, and well established by 8 d postinfection, at which point a clear difference was seen. Virus expressing UL141 demonstrated significantly lower formation of large multinucleated cells in comparison with virus lacking UL141 (Fig. 6). Furthermore, the size of syncytia was much lower in wildtype virus as compared to the virus lacking UL141 expression. Deletion of the ER retention domain resulted in syncytia of comparable size and number to ΔUL141 virus, indicating that this phenomenon was due to reduced cell-surface gH-complex trafficking as opposed to the presence of gpUL141 in cell surface complexes. Thus, gpUL141-mediated modulation of entry glycoproteins inhibits syncytium formation.

### gpUL141-Mediated Modulation of gH-Complex Levels Inhibits Humoral Antiviral Functions.

**Fig. 7.**
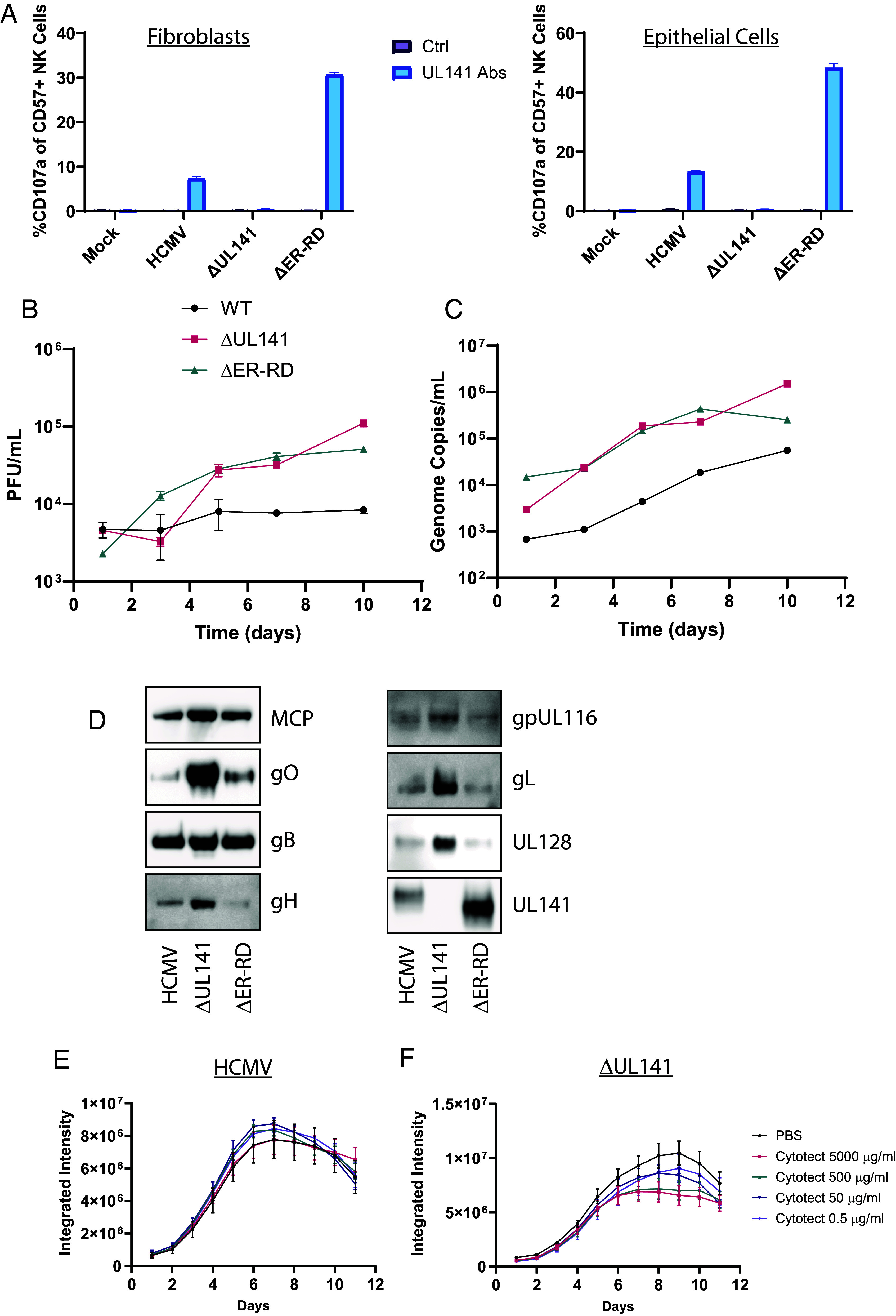
gpUL141 retention of gH-complexes alters virus growth, virion incorporation, and susceptibility to neutralizing antibodies. (*A*) HFFF were infected for 96 h, alternatively HFFF were infected for 72 h and then cocultured with RPE-1 cells for 24 h before infected RPE-1 cells were isolated by MACs, and left until 96 h post coculture. Infected cells were then cocultured with NK cells for 5 h in the presence of CD107a antibody and golgistop, before the percentage of degranulating NK cells (i.e., those demonstrating ADCC) were assessed. (*B* and *C*) HFFF were infected for 72 h at high MOI with wildtype HCMV strain Merlin-GFP or virus lacking UL141 then cocultured with uninfected RPE-1 cells. 5d later the supernatants were harvested and analyzed for live virus by plaque assay (*B*) or encapsidated genomes by qPCR (*C*). (*D*) HFFF were infected for 72 h with wildtype HCMV strain Merlin or virus lacking either the whole of gpUL141, or just the C-terminal intracellular portion of gpUL141, which contains a ER-retention domain (ER-RD). HFFF were cocultured with RPE-1 cells and maintained until 100% CPE, at which point cell-free virions were purified and analyzed by western blot. (*E* and *F*) HFFF were infected for 72 h with HCMV strain Merlin-GFP or the same virus lacking gpUL141, then added to uninfected HUVEC. 5 d later the indicated concentrations of cytotect were added, and virus dissemination over time assessed by Incucyte.

Cell-surface viral antigens are a target for humoral immunity, with opsonized HCMV infected cells susceptible to NK-mediated attack through antibody-dependent cellular cytotoxicity (ADCC). Antibodies capable of mediating ADCC against gB or gH-containing complexes have not been described, therefore to determine whether greater trafficking of antigens to the cell surface can enhance susceptibility to ADCC, we tested levels of ADCC induced by antibodies that we have previously isolated against gpUL141 itself. ADCC was significantly enhanced by the greater cell-surface trafficking that arose from loss of the ER retention domain on UL141 (Fig. 7*A*).

To determine whether the ER retention domain also altered the growth properties of virus, we assessed cell-free virus titers in epithelial cells. As previously, complete loss of UL141 led to higher cell-free titers, and this was phenocopied by the loss of just the ER retention domain (Fig. 7*B*). This was due to a physical loss of virion secretion because qPCR of viral genomes from the supernatant also demonstrated reductions (Fig. 7*C*). Surprisingly, when the genome:PFU ratio at day 7 was calculated, values of 2.4, 7.2, and 10.6 for WT, ΔUL141, and ΔER-RD respectively, indicate that although UL141 reduced particle release, its presence in wildtype virus led to slightly higher particle infectivity, while viruses lacking UL141 demonstrated lower infectivity. Purification of virions demonstrated that, as in earlier data (Fig. 3*H*), loss of gpUL141 led to increases in levels of all gpUL141-interacting proteins, with loss of just the ER retention domain resulting in increased levels of some gH-complex containing proteins, with gO in particular restored (Fig. 7*D*). Thus, the alteration in growth properties associated with gpUL141 expression arises from the presence of the ER retention domain, which also modulates some virion glycoprotein levels in the virion.

We have previously found that modulating the expression levels of gH-containing complexes alters the sensitivity of HCMV to neutralizing antibodies during spread within a monolayer (15). To determine whether the changes seen here had a similar impact, we infected cells at low MOI with GFP-expressing HCMV strains that either contained or lacked UL141, and measured dissemination over time in the presence of Cytotect, a pooled polyclonal preparation of antibodies from HCMV seropositive individuals and selected for high neutralizing activity. Whereas wildtype virus was completely resistant to neutralizing antibodies, virus lacking UL141 was susceptible (Fig. 7*E*).

Therefore, gpUL141 contains an ER retention domain which is not required for it to modulate ligands for activating receptors on NK cells, but acts to restrict cell-surface expression of gB and all gH-containing glycoprotein complexes, while also affecting virion envelope composition. This reduces virus dissemination, but renders it more resistant to both neutralizing antibodies and ADCC. Thus, UL141 acts as an evasin of both innate (NK) and adaptive (humoral) immunity.

## Discussion

Our analysis significantly expands the repertoire of proteins found in the HCMV virion, adding at least 18 viral proteins found in the majority of highly purified preparations, at equal or greater abundance than established components. Not all detected proteins need be packaged specifically in the virion, cellular RNAs for example can be packaged in virions in proportion to their relative abundance, indicating a nonspecific process (53). Nevertheless, a significant number of cellular proteins were enriched in the virion, implying their specific recruitment either to the virion itself or to sites of virion assembly. Furthermore, gpUL141 provides an exemplar of the novel viral proteins identified, demonstrating that they can be bona fide components that play key functional roles in virion assembly and function.

gpUL141 was previously recognized to promote virus survival through three distinct mechanisms [targeting CD155 (14), CD112 (13), all four TRAIL receptors (12)]. As cell and virion surface proteins, there are multiple examples of viral entry glycoproteins being “co-opted” to also act as immune evasins (54). However, although gpUL141 is found in the virion in complex with nearly all HCMV entry glycoprotein complexes, our data argue that rather than acting in entry, gpUL141 acts to modulate the levels of other glycoproteins in order to control syncytium formation and avoid both neutralizing and nonneutralizing antibody-mediated control. Given that gpUL141 interacts with multiple host cell-surface proteins, a potential function for virion-presented gpUL141 could involve receptor-mediated viral entry. However, given that the presence of gpUL141 results in reduced virus dissemination and lower cell-free titers in epithelial and endothelial cells in vitro, any such functions do not seem to be proviral. Furthermore, the ability of gpUL141 to restrict cell-free virus release was lost when the ER retention domain was removed, implying that this phenotype arises from influences on glycoprotein trafficking rather than receptor binding. It is also notable that the structural model indicates that gpUL141 cannot bind to both viral (gH) and cellular (TRAIL-R2) factors simultaneously. Nevertheless, the presence of gpUL141 in the virion does have the potential to influence virus biology through the dimerization revealed by the structural models. Although gpUL141 reduced virus particle release and envelope glycoprotein incorporation, it did not reduce per-particle infectiousness. It is possible that dimerization of entry complexes increases avidity to compensate for reductions in abundance. Furthermore, since UL141 occludes at least one neutralizing epitope on gH (49) incorporation into the virion may directly influence epitope-specific immunity.

As a large and complex virus with a broad tropism, HCMV encodes over 10 different envelope glycoproteins that form the core receptor binding and envelope fusion functions (55). gB transits via the plasma membrane before being internalized to be delivered to the viral assembly complex (VAC) for virion incorporation (56), and presumably the same is true for other glycoproteins. All are therefore potential targets for ADCC. Through targeting two viral proteins (gH and gB) that are common to nearly all of these complexes, gpUL141 provides an elegant solution to this problem. Both knock-in (RAd) and knock-out (HCMV; both whole protein and ER-RD) support a model in which gpUL141 inhibits plasma-membrane transport and subsequent virion incorporation of the majority of HCMV entry glycoproteins. We have previously shown that the humoral ADCC response during natural infection is highly polyclonal, targeting numerous proteins from the early phase of the viral lifecycle onwards (10). ADCC activity peaked prior to the expression of entry glycoproteins. This could be in part due to UL141 limiting glycoprotein levels, however it is also possible that UL141-mediated intracellular retention is important to limit the generation of ADCC-antibodies against entry glycoproteins during infection. Dissecting this will require isolating and mapping monoclonal antibodies that target gB and Trimer/Pentamer/gpUL116 that are ADCC-capable, as we have previously for gpUL141. However, given the large number of antigens that gpUL141 influences, even small alterations in cell-surface levels have the potential to produce significant effects.

Although complete loss of gpUL141 resulted in robust effects on plasma membrane and virion envelope composition, the impact of removing just the ER-RD was more varied. Modulation of plasma membrane levels were either partially or entirely dependent on the ER-RD, depending on the assay and protein being tested. This likely reflects the presence of multiple different competing signals within the C-terminal intracellular domain of gpUL141, all of which were lost by complete deletion. In addition to the ER retention dilysine motif in its C-terminus, gpUL141 also encodes a Yxxϕ motif for endosomal retrieval from the plasma membrane (57). ER trafficking can also depend on interactions between C-terminal and membrane domains (58). Thus, gpUL141 may both limit ER-exit of complexes while also promoting reinternalization into the VAC of those that do traffic to the cell surface. Loss of both motifs may influence multiple processes, with the final outcome also depending on the motifs found in component proteins of the different complexes—for example gB has its own retrieval motif (56). Assessing this will require more in-depth analysis and targeted mutagenesis of the gpUL141 cytoplasmic tail.

Despite the partial effects seen biochemically following ER-RD deletion, effects on levels of cell-free virus were almost entirely recovered. This may indicate a threshold effect, in which levels are restored sufficiently to improve virus release—in particular gO, which is required for cell-free infection, was upregulated in this virus. These assays imply that for HCMV, reducing envelope glycoprotein levels in the virion may impact virus release, but this is a price worth paying if it also results in lower cell-surface antigenic targets for ADCC, resistance to neutralizing antibodies, and lower syncytium formation. The role of syncytia in vivo is unclear, however HCMV strains that lack UL141 form syncytia in a range of cell types in vitro (59–62). It is clear from these studies that syncytium formation depends on both cell type and genetic variants of viral proteins including gB (63, 64). Our data imply that syncytia may be detrimental to HCMV in vivo since UL141 specifically reduces it. However, although UL141 may modulate the extent of these effects, it is unlikely to completely prevent them since we still saw small syncytia (~2 nuclei) during infection of ARPE19 cells with wildtype virus.

The levels of multiple envelope glycoprotein complexes are notably different in Merlin compared to passaged HCMV strains. As a ratio, Merlin has higher levels of pentamer and lower levels of trimer (32, 33), and expresses gpRL13 (8). At least some of these differences arise from in vitro acquired mutations in passaged strains (8, 32); whether they can also arise from genuine strain-specific effects remains to be determined. Nevertheless, they result in Merlin disseminating via a form of cell–cell spread that is exceptionally resistant to neutralizing antibodies (8, 15). Subsequent studies have shown that although these differences explain some of the antibody-resistance of Merlin, they are not the only factor (65); gpUL141 provides an explanation for these observations. There may also be interplay between gpUL141 and other viral proteins that modulate glycoprotein trafficking. UL148 leads to reduction of pentamer levels in the virion in favor of trimer (66) by preventing gO degradation (67), while gpUL116 acts as a chaperone for gH to get to the cell surface (41, 42) and trimer incorporation into the virion (40). Further work will be needed to investigate these impacts in the presence of UL141.

Our work also explains the long-standing observation that the UL140-145 genome region is frequently lost when HCMV is passaged in vitro. Mutations adjacent-to or involving UL141 have been seen in clinical strains passaged in fibroblast, epithelial, and endothelial cells (6), when Merlin was passaged in epithelial cells (37), and in multiple laboratory strains including TB40 (14, 68), VR1814, and the derived FIX-BAC (6), and all AD169 variants (69), many of which were fibroblast-derived. UL141 is clearly detrimental to virus release and dissemination in epithelial and endothelial cells, however these studies imply that it may also be detrimental in fibroblasts. No growth phenotype was associated with UL141 in Merlin in fibroblasts, possibly because the effects on glycoprotein trafficking were weaker than in epithelial cells. Nevertheless, gpUL141 can clearly reduce glycoprotein incorporation into the virion in fibroblasts. gO is hypervariable (70, 71) and gO variants influence other glycoproteins as well as virus growth in diverse ways (33, 72–74). Therefore, different phenotypes may be observed when viruses encode other gO genotypes.

Overall, we have defined the proteome of a wildtype HCMV virion, and demonstrate that novel components encoded in clinical virus have important biological effects. One of these (gpUL141) represents a novel viral strategy for immune evasion which impacts numerous aspects of the lifecycle that are relevant to therapeutic and vaccine design. It is therefore important that genetically intact HCMV strains are tested during preclinical development.

## Methods

### Cell Culture.

Human fetal foreskin fibroblasts (HFFFs) immortalized with human telomerase reverse transcriptase (HF-TERTs), HF-TERT-immortalized HFFFs expressing Coxsackie adenovirus receptor (HF-CAR) (15), retinal pigment epithelial-1 (RPE-1 and ARPE19) cells were maintained under standard conditions in DMEM (Sigma) supplemented with 10% heat-inactivated fetal bovine serum (Sigma). Human umbilical vein endothelial cells (HUVEC) were maintained under standard conditions in Endothelial Cell Growth Medium (Basal medium plus individual supplements; PromoCell). All human cell lines were deidentified prior to use.

### Viruses and Infections.

HCMV variants were recovered from an infectious bacterial artificial chromosome (BAC) clone of strain Merlin. This encodes the complete strain Merlin genome, which was repaired to match the sequence of the original clinical material (8). Mutations are rapidly selected in the genes encoding RL13 and UL128L upon passage in vitro, therefore the promoters for these genes have been rendered responsive to the tetracycline repressor (TetR). In cells expressing tetR, expression is restricted, allowing recovery of genetically intact virus (37). This virus loses tropism for cell types other than fibroblasts due to repression of UL128L, but can readily infect fibroblasts. If these fibroblasts lack tetR then UL128L is expressed, and virus can be transferred to other cell types (e.g., endothelial and epithelial cells) by coculture. Deletion of the UL141 ER retention domain was carried out by en-passant mutagenesis as described previously (8). Replication deficient Adenovirus vectors were constructed in the AdZ system as previously described (75). All modifications were verified by Sanger sequencing of the modified site, and all viruses underwent whole genome sequencing following recovery from the BAC (37). Cell-free titers were determined using an IE1 microplaque assay.

For proteomics and biochemical analysis cell-free virus was harvested from the supernatant, cell debris removed by low-speed centrifugation (420×*g*, 3 min), then samples concentrated either by dialysis (Viva flow 50R, Sartorius, 1MDa cut-off) or by pelleting through a 20% sorbitol cushion (82,700×*g*, 1 h). Intact virions were then separated from cellular debris, dense bodies, and noninfectious particles by positive density/negative viscosity gradient centrifugation, as previously described (76).

Unless otherwise stated HCMV infection of HF-Terts was at MOI = 5 for 72 h. To infect RPE-1 or HUVEC, HF-Terts infected for 72 h were cocultured with target cells at ratios of 0.03:1, 1:2, or 4:1 HF-Tert:RPE-1/HUVEC for virus dissemination and syncytia analysis, growth kinetics, or PNGase F assays, respectively. For dissemination inhibition assays in HUVEC, Cytotect® was added to cells at 3 d post coculture in a 10-fold serial dilution starting at 5,000 µg/mL. HF-CARs were infected with RAd at MOIs stated, for 48 h.

### Statistics.

All statistical analyses were carried out in GraphPad Prism v10.4.0. All assays were carried out in a minimum of technical triplicate, and repeated at least three times. Statistical tests carried out for each assay are noted in figure legends.

Further experimental methods can be found in *SI Appendix*.

## Supplementary Material

Appendix 01 (PDF)

Dataset S01 (TXT)

Dataset S02 (XLSX)

Dataset S03 (TXT)

## Data Availability

Alphafold Modelling data have been deposited in Zenodo (https://doi.org/10.5281/zenodo.16351176) (77).
