# Supplementary material for: Virion proteomics of genetically intact HCMV reveals a regulator of envelope glycoprotein composition that protects against humoral immunity
Source: Proc Natl Acad Sci U S A. 2025 Sep 18;122(38):e2425622122. doi: 10.1073/pnas.2425622122 (PMC12478159; doi:10.1073/pnas.2425622122)
Supplement: Supplementary file 1 — Appendix 01 (PDF) [file pnas.2425622122.sapp.pdf]

PNAS

Supporting Information for:
Virion Proteomics of Genetically Intact HCMV Reveals a Regulator of Envelope Glycoprotein Composition that Protects Against Humoral Immunity .

Kirsten Bentley[1]#, Evelina Statkute[1]#, Isa Murrell[1]#, Ceri A. Fielding[1], Robin Antrobus[2], Hannah Preston[1], Lauren Kerr-Jones[1], Daniel Cochrane[1], Ilija Brizic[3], Paul J. Lehner[4], Gavin W.G. Wilkinson[1], Eddie C.Y. Wang[1], Stephen C. Graham[5], Michael P. Weekes[2], Richard J. Stanton[1]*

#These authors contributed equally
1. Division of Infection and Immunity, Cardiff University School of Medicine, Cardiff, CF14 4XN
2. Cambridge University Institute for Medical Research, Cambridge University, Cambridge CB2 0XY
3. Center for Proteomics, School of Medicine, University of Rijeka, 51000 Rijeka, Croatia
4. Cambridge Institute for Therapeutic Immunology and Infectious Disease, Cambridge University, Cambridge CB2 0AW
5. Department of Pathology, University of Cambridge, Cambridge, CB2 1QP

Corresponding Author
StantonRJ@cardiff.ac.uk

This PDF file includes:

Extended Methods
Figures S1 to S4
Table S1
Legends for Datasets S1 to S3
SI References

Other supporting materials for this manuscript include the following:

Datasets S1 to S3

Supporting Information Text

Methods

Viruses and Infections

Deletion of the UL141 ER retention domain in the strain Merlin BAC was carried out by en-passant mutagenesis[1] with primers 5'-ACACACTGCATTTTTTAACATCTTATTTTTTTATTTTATGCGTGTTCTCAACAGCACTGCAGGTAACACATAGGGATAACAGGGTAATCGATTT-3' and 5'-TTTTGGAGTATTTTCACCGTATGTTTCCTATGCTACCTGTGTTACCTGCAGTGCTGTTGAGAACACGCATAAAATAAGCCAGTGTTACAACCAATTAACC-3'. A V5 epitope tag was added to the C-terminus of UL141 by recombineering as previously described[2], using primers 5'-AGGGGACGACGAGGCGGTGAGGGCTATCGACGCCTACCGACTTACGATAGTTACCCCGGTGTTAAAAAGATGAAGAGGCCTGTGACGGAAGATCACTTCG-3' and 5'-GCATATTTTAATCACACTATTCACATTTCACACACTGCATTTTTTAACATCTTATTTTTTTATTTTATGCGTGTTCTCACTGAGGTTCTTATGGCTCTTG-3' to insert the SacB cassette and primers 5'-GACGCCTACCGACTTACGATAGTTACCCCGGTGTTAAAAAGATGAAGAGGGGCTCCGGGGGGTCGGGTGGAAGTGGCGGTAAGCCAATCCCTAACCCGCT-3' and 5'-ACACACTGCATTTTTTAACATCTTATTTTTTTATTTTATGCGTGTTCTCACGTAGAATCAAGACCTAGGAGCGGGTTAGGGATTGGCTTACCGCCACTTC-3' to replace the cassette with a V5 epitope tag. A HA tag was added to the C-terminus of UL116 by en-passant mutagenesis using primers 5'-ACCTGAGTGCCAACTTTTGGCGCCAACTGGCTCCTTACCGTCACACTCTCATCGTGCCGCAGACTAGCGCTTACCCCTACGACGTGCCCGACTACGCCTG-3' and 5'-CAACACCACAGCAGTATCACCGGTCCAGGTGAGAAAGAGAAGCCGCAATCCGGGCGGCGGCACATCAGGCGTAGTCGGGCACGTCGTAGGGGTAAGCGCT-3'. All modifications were verified by Sanger sequencing of the modified site, and all viruses underwent whole genome sequencing following recovery from the BAC[3].

Infectious HCMV was recovered by transfecting HF-Tet cells with an Amaxa Nucleofector, the basic fibroblast kit, and program T-16. Virus was harvested from the supernatant when 100% CPE was achieved, then cells remove by low speed centrifugation (420 x*g*, 3 min), before virus was pelleted by high speed centrifugation (29,500 x*g*, 2h). Virus was resuspended in DMEM containing 10% FCS, aliquoted, and stored at -80°C. Cell-free titres were determined using an IE1 micro plaque assay. Briefly, HF-Terts were seeded in 96-well plates 18 h prior to use. Virus supernatants were 10-fold serially diluted in DMEM-FBS and added to cells. At 24 h p.i. media was removed and cells fixed in ice cold 50:50 Acetone/Methanol for 15 min. Cells were washed once in PBS and primary antibody added at 1:1000 in PBS for 30 min at 37°C. Cells were washed once in PBS and secondary antibody added at 1:500 in PBS for 30 min at 37°C, followed by a final wash in PBS. Infected cells were counted using an Incucyte® (see below).

Proteomics

For analysis of virions by MS, cells were passaged for two weeks in SILAC-DMEM containing arginine and lysine in either their normal, medium (13C6), or heavy (13C6-15N4, 13C6 15N2 respectively) forms respectively, before being infected and virus harvested and purified as above. Following purification samples were lysed in LDS sample buffer and run 1.5cm into a Bis-Tris Midi Gel (Thermo), before lanes were excised with a clean scalpel and cut into 6 equally sized bands. Samples were reduced, alkylated and digested in-gel using trypsin. Digested peptides were eluted with MeCN/water and 5% FA washes. Peptides were pooled in 0.5ml tubes (Protein LoBind, Eppendorf) and dried almost to completion. Samples were re-suspended in 15 µl solvent (3% MeCN, 0.1% TFA) with 7µl analysed by LC-MSMS using a Thermo Q Exactive mass spectrometer (Thermo Fischer Scientific) equipped with an EASYspray source and coupled to an RSLC3000 nano UPLC (Thermo Fischer Scientific). Peptides were fractionated using a 50cm C18 PepMap EASYspray column maintained at 35°C with a solvent flow rate of 250nl/min. A gradient was formed using solvent A (0.1% formic acid) and solvent B (80% acetonitrile, 0.1% formic acid) rising from 3% to 40% solvent B by 90 min followed by a 4 min wash at 95% solvent B. MS spectra were acquired at 70,000 resolution between m/z 400 and 1650 with MSMS spectra acquired at 17,500 fwhm following HCD

activation. Data was processed in Maxquant 2.4.8.0 with carbamidomethyl (C) set as a fixed modification and oxidation (M) and acetyl (protein N-terminus) set as variable modifications. Data was searched against a Uniprot Homo Sapien database (downloaded 27/05/14) a curated database of canonical HCMV proteins and a database of common contaminants.

Separation of Subpopulations Following Co-culture

Where it was necessary to separate cell types following virus transfer by co-culture (e.g. for CD107a assays), a previously established magnetic-activated cell sorting (MACS) separation protocol was used[4]. In brief, HFFF expressing His-tagged mCherry protein on their cell surface were infected with HCMV for 72h, then co-cultured with target cells. Twenty-four hours post co-culture cell monolayers were washed and detached, stained with anti-His antibody (Antibody and Reagents Table) followed by anti-mouse-IgG magnetic beads (Miltenyi Biotec), and separated by MACS (Miltenyi Biotec). Newly infected RPE-1 or HUVEC cells were maintained for a further 48 h prior to CD107a assay.

Immunoprecipitation

Cells or viruses were lysed in IP Lysis Buffer (Pierce) for 10 min in the presence of protease inhibitors (Sigma), then nuclei removed by centrifugation. Lysates were incubated with anti-V5 or anti-HA agarose beads (Abcam) overnight, before being washed 3 times in lysis buffer and proteins eluted by boiling for 10 min in 4X LDS sample buffer (Thermo Fisher).

PNGaseF and EndoH digestion

Samples were digested according to manufacturers instructions (NEB). In brief, samples were lysed in denaturing buffer then the appropriate reaction buffer added, and incubated overnight at 37°C in the presence of enzyme. Samples without enzyme added served as controls. The next day LDS sample buffer was added and samples boiled for 10 min before being analysed by SDS-PAGE and western blot.

Western blot

Samples were lysed in 4X LDS sample buffer (Thermo Fisher) then boiled for 10 min before being loaded onto Bis-Tris Midi gels (Thermo Fisher) and run for 1 h at 200V. Following separation samples were transferred onto PVDF (Amersham) by semi-dry blotting, then blocked in 5% Milk/PBST (blocking buffer) for 1 h. Primary antibodies were added in blocking buffer for 1 h at room temperature or overnight at 4°C, washed 3x in PBST, then secondary HRP-conjugated antibodies added in blocking buffer for 1 h. After washing 3x in PBST membranes were incubated with supersignal west pico (Thermo Fisher) and imaged on a Syngene XX6 geldoc.

CD107a Assay

PBMCs were isolated from healthy donors and rested overnight in RPMI supplemented with 10% FCS, and L-glutamine (2 mM). Target cells were harvested using TrypLE Express (Thermo Fisher), then mixed with PBMC at an effector:target ratio of 10:1 in the presence of golgistop (0.7 µl/ml, BD Biosciences) anti-CD107a–FITC (clone H4A3, BioLegend), and relevant anti-HCMV antibodies. Cells were incubated for 5 h at 37°C, washed in cold PBS, and stained with live/dead Fixable Aqua (Thermo Fisher), anti-CD3–PECy7 (clone UCHT1, BioLegend), anti-CD56–BV605 (clone 5.1H11, BioLegend), anti-CD57–APC (clone HNK-1, BioLegend). Data were acquired using an Attune NxT Flow Cytometer (Thermo Fisher) and analysed with FlowJo software version 10 (FlowJo LLC).

Flow Cytometry

Cells were washed in PBS, dissociated with TrypLE Express (Thermo Fisher) and stained with primary antibody (Antibody and Reagents Table) for 30 min at 4°C. Cells were washed in PBS and incubated with the secondary antibody for 30 min at 4°C before being washed and fixed with 4% paraformaldehyde (PFA).

All data was acquired using an Attune Nxt Flow Cytometer (Thermo Fisher), and analysed using FlowJo Software version 10 (FlowJo LLC).

qPCR

Viral DNA was extracted from DNase I treated (RQ1 RNase-free DNase; Promega) supernatants with the QIAamp MinElute Virus Spin Kit (Qiagen) as per manufacturers' instructions. Quantitative PCR reactions were performed in a Applied Biosystems Quant Studio 3 Thermal Cycler (Thermo Fisher Scientific). Reactions were set up using FastGene 2x IC Green qPCR Universal Mix (Nippon Genetics; Geneflow), 0.4 µM of forward and reverse primers, and 100 ng of cDNA in a final volume of 20 µL. The gB forward primer was 5′-CTGCGTGATATGAACGTGAAGG-3′ and the reverse primer was 5′-ACTGCACGTACGAGCTGTTGG-3′. Amplification was performed using the Comparative cT with Melt programme (2 min at 50°C, 10 min at 95°C, followed by 40 cycles of 15 sec at 95°C and 1 min at 60°C. Melt curve: 15 sec at 95°C, 1 min at 60°C and 15 sec at 95°C). Water-only negative controls and a serial dilution of a positive control standard (plasmid containing gB) were included in each run. CMV genome equivalents were calculated from the Ct values generated in The Standard Curve App (Thermo Fisher Scientific Connect Data Analysis Apps).

Incucyte®

Virus dissemination assays and virus titrations were measured on an Incucyte® SX5 Live Cell Analysis System, scanning the whole well with a 4X lens, with both brightfield and green image channels. Wells were scanned every 24 h for 10 days for dissemination and neutralisation assays, or one-off scans for titrations. Incucyte® Basic Analysis Software was used to measure the Total Integrated Intensity (GCU x μ^2/well) for time course analyses, or Count (per well) for titrations.

Syncytia Imaging and Analysis

Images were taken from 10 randomly selected fields of view at 20X magnification on a Zeiss Apotome 2 microscope. Syncytia were determined by the presence of two or more nuclei observable within a single cell membrane. Cell area was measured using Zeiss Zen Lite v3.10 software and nuclei counted manually. The average area of fluorescence within syncytia was expressed as a percentage of the total fluorescent cell area.

Structure prediction

The structures of gH (UniProt Q6SW67 residues 25–719) in complex with gpUL141 (UniProt Q6RJQ3 residues 37–278) and gpUL116 (UniProt Q6SW34 residues 25–313), or of a trimer of gB (UniProt F5HB53 residues 25–751) in complex were predicted using AlphaFold3[5]. For all, predicted signal sequences and transmembrane regions were excluded from the sequence used for structure prediction. Structures were superposed and molecular images were generated using PyMOL (Schrödinger) or COOT[6].

Data availability

Atomic coordinates and per-residue quality statistics for the AlphaFold3 models have been deposited in the University of Cambridge Apollo repository (DOI: 10.17863/CAM.118380) and Zenodo (10.5281/zenodo.16351176). Raw proteomics files are available at PRiDE (DOI:PXD065223).

Antibody and Reagents Table

**Reagent**	**Source**	**Identifier**
Primary antibodies for flow cytometry		
Mouse anti-CD155 (clone D171)	Invitrogen	MA5-13493
Mouse anti-CD112 (clone b-c12)	Santa Cruz	sc-65333

Mouse anti-TRAIL-R2 (human; clone HS201)	AdipoGen Life Sciences	AG-20B-0023
Mouse-anti CMV gH	BioRad	2470-5437
Mouse anti-gB (SM5-MsFC)	In-house	
Primary antibodies for immunoblotting		
Mouse anti-CMV gB (clone 2F12)	Abcam	ab6499
Mouse anti-CMV (IE1)	Merck	MAB810R
Mouse anti-gpUL141 (clones M550.2 and M550.3)	In house[7]	
Mouse anti-MCP (clone 28-4)	A gift from William Britt, University of Alabama, USA.	
Rabbit anti-gH	A gift from Brent Ryckman, University of Montana[8]	
Rabbit anti-gL	A gift from Brent Ryckman, University of Montana[8]	
Mouse anti-gO	A gift from Stipan Jonjic[9]	
Mouse anti-gpUL116	Gift from Marcello Merona, University at Naples, Italy[10]	
Mouse anti-gpUL128	A gift from Giuseppe Gerna[11]	
Mouse anti-human HLA-A/B/C (clone W6/32)	BioLegend	311402
Mouse anti-V5 tag	BioRad	MCA1360
Rabbit anti-V5	Abcam	ab9116
Anti-HA Tag (clone 11A3A05)	BioLegend	660002
Rabbit Anti-Actin	Sigma	A2066
Antibodies for CD107a assay		
Mouse FITC anti-human CD107a	BioLegend	328606
Mouse PE/Cy7 anti-human CD3	BioLegend	300420
Mouse APC anti-human CD57	BioLegend	359610
Mouse BV605 anti-human CD56	BioLegend	362538
Primary antibody for titration		
Mouse anti-CMV (IE1)	Merck	MAB810R
Secondary antibodies		
Goat anti-mouse HRP	BioRad	170-6516
Goat anti-rabbit HRP	BioRad	170-6515
Goat anti-mouse Alexa Fluor 488	Invitrogen	A11017
Goat anti-mouse Alexa Fluor 647	Invitrogen	A21235

Goat anti-rabbit Alexa Fluor 647	Invitrogen	A21246
Antibodies and Reagents for MACS Separation		
Mouse anti-His (clone His.H8)	Thermo Fisher	MA1-21315
MACS anti-IgG MicroBeads	Miltenyi Biotec	130-048-401
MACS LS Columns	Miltenyi Biotec	130-042-401
MACS Separation Buffer	Miltenyi Biotec	130-091-221
MACS Pre-Separation Filters (70μM)	Miltenyi Biotec	130-095-823
Additional Reagents		
Aqua live/dead	Thermo Fisher	L34966
GolgiStop	BD Biosciences	554724
PNGase F	NEB	P0704
Endo H	NEB	P0702
Agarose anti-V5 tag	Abcam	ab1229
Agarose anti-HA tag	Abcam	ab214758
FastGene 2x IC Green qPCR Universal Mix	Geneflow	P8-0058
CellBrite® Steady 550 Membrane Staining Kit	biotium	30107

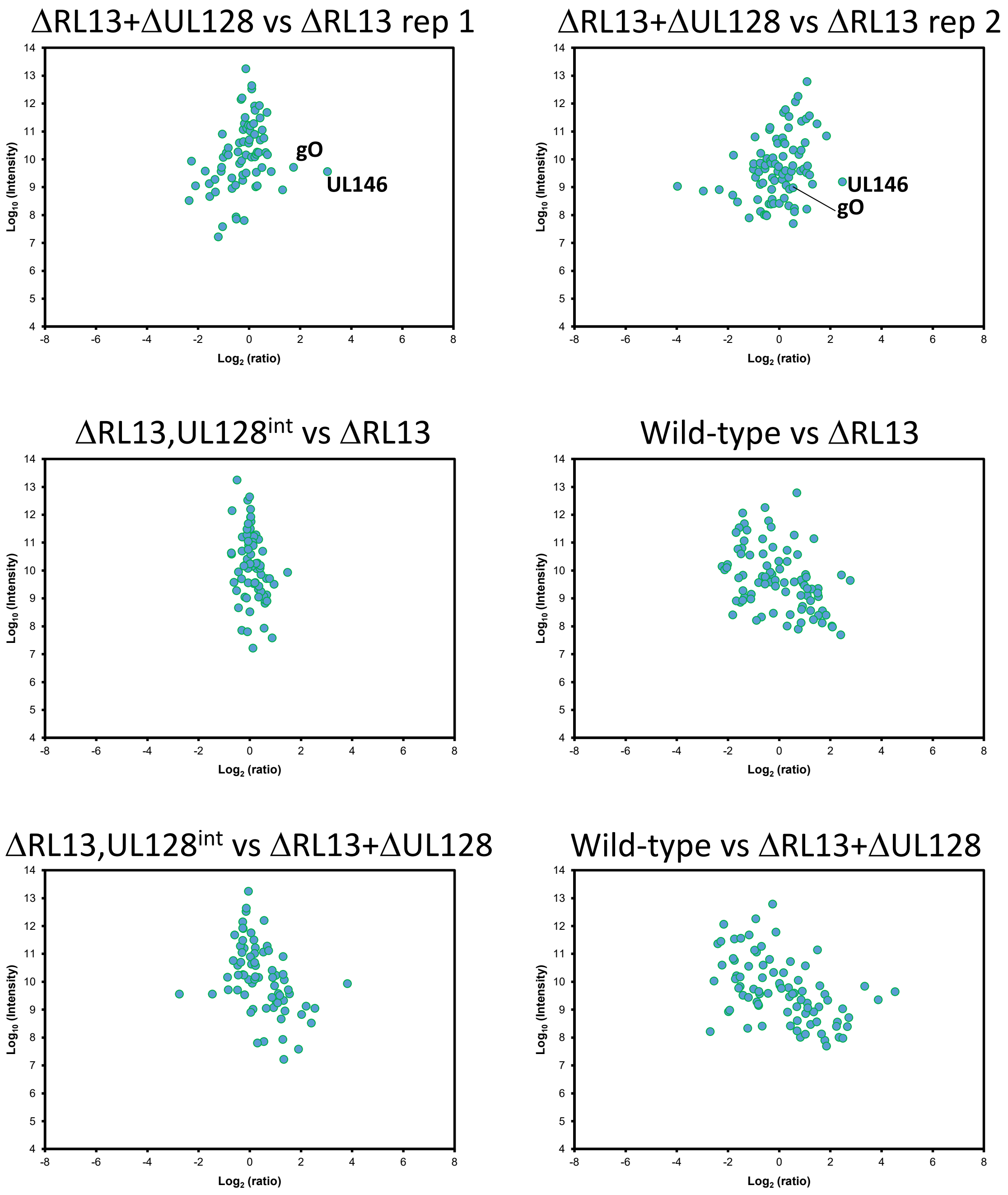

Fig. S1. Scatter plot of viral proteins quantified in each SILAC-labelled virion experiment. The summed ion intensity (y axis) is shown as log10, and ratio (x-axis) as log2.

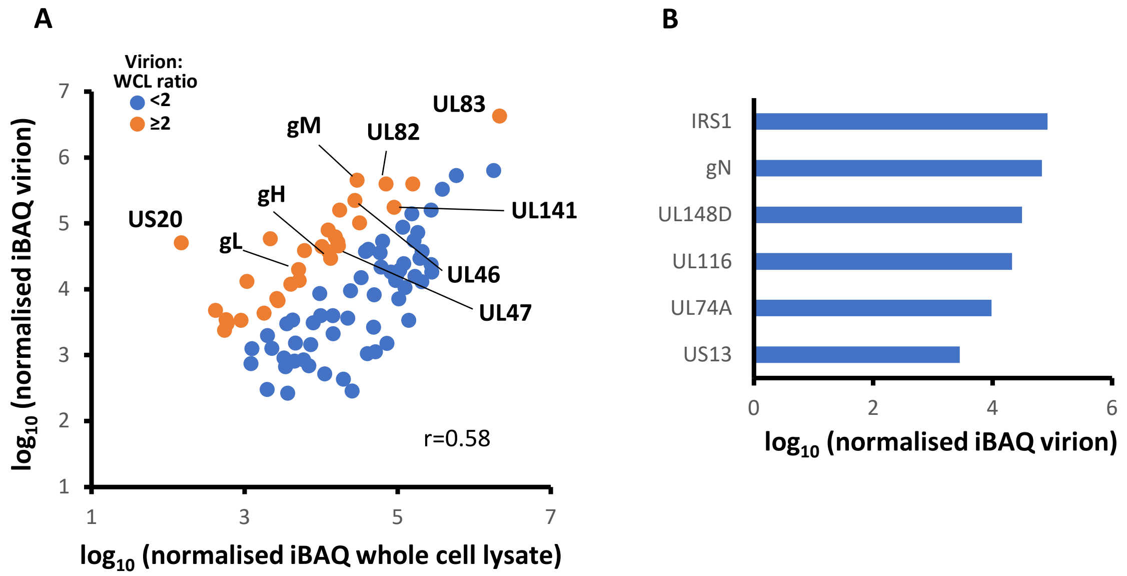

Fig. S2. (A) comparison of abundance of HCMV proteins in the virion and in whole cell lysates (WCL) of infected cells. Proteins displayed in orange had a virion:WCL ratio of >2. (B) HCMV proteins only detected in the virion but not in WCL of infected cells. All quantified proteins are shown in Dataset S2.

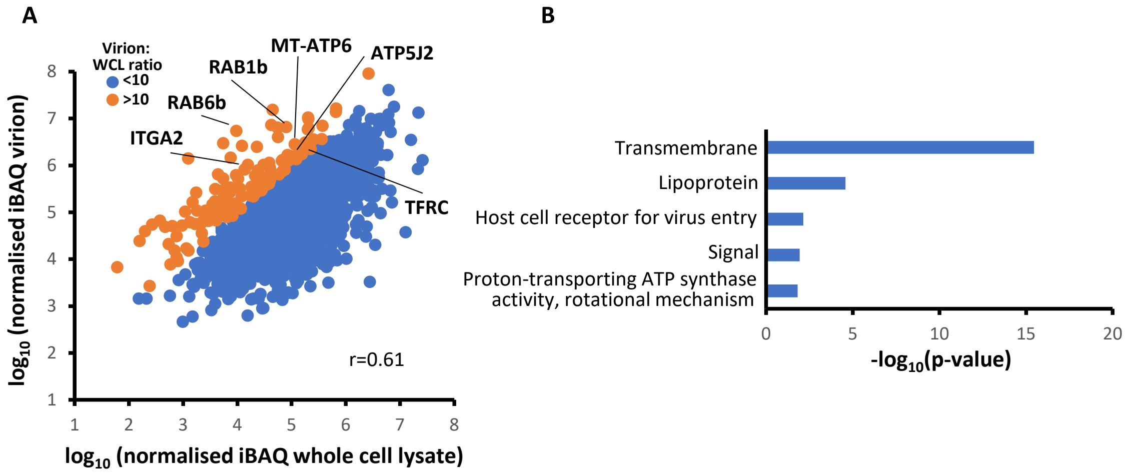

Fig. S3. (A) comparison of abundance of human proteins in the virion and in whole cell lysates (WCL) of infected cells. Proteins displayed in orange had a virion:WCL ratio of >10. All quantified proteins are shown in Dataset S2B. (B) Enrichment of pathways within human proteins with a virion:WCL ratio of >2, using DAVID software, in comparison to all quantified human proteins. Full data are shown in Dataset S2C.

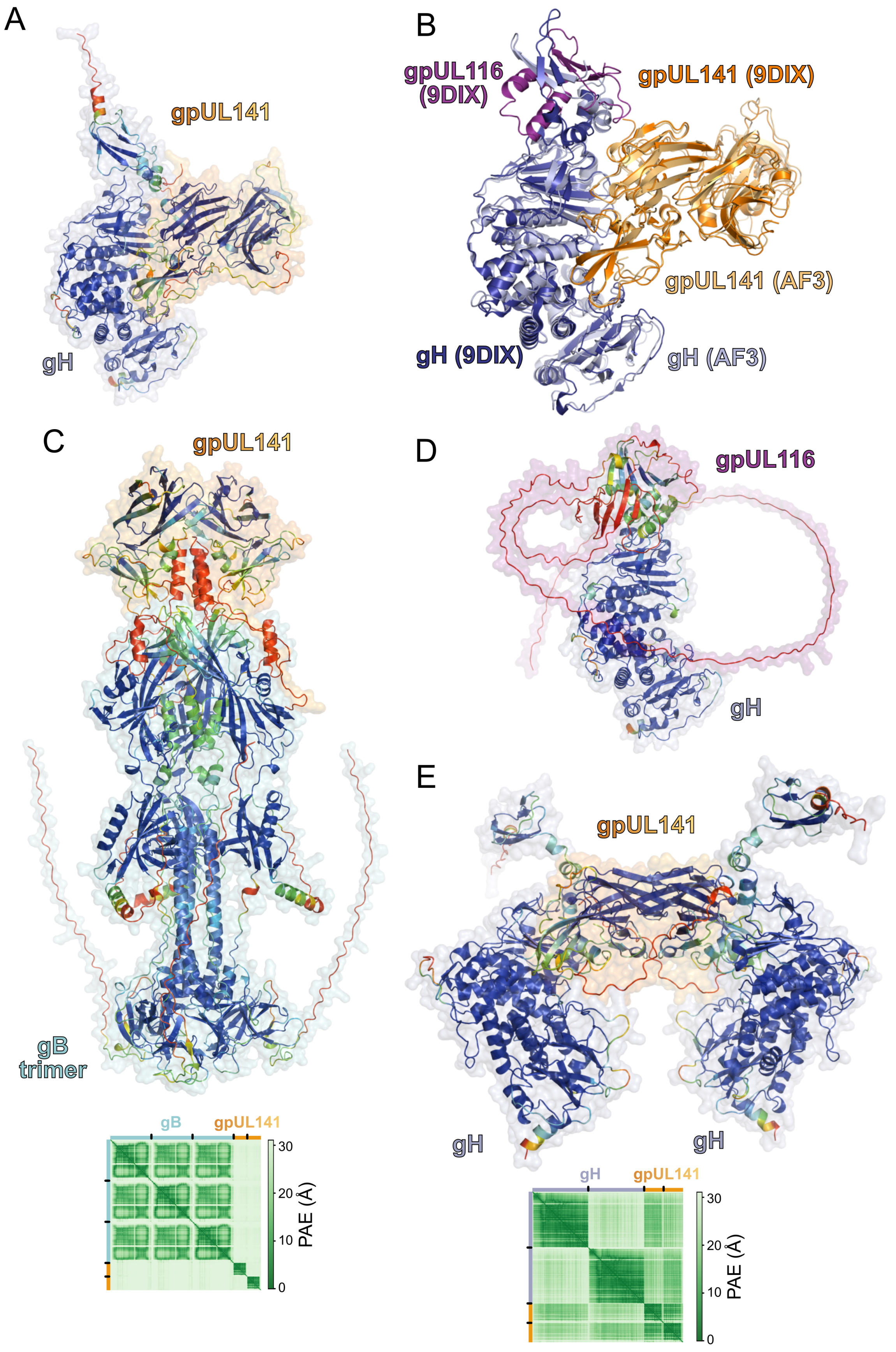
A
gpUL141
gH
B
gpUL116
(9DIX)
gpUL141 (9DIX)
gpUL141 (AF3)
gH (9DIX)
gH (AF3)
C
gpUL141
gB
trimer
gB
gpUL141
PAE (Å)
30
20
10
0
D
gpUL116
gH
E
gpUL141
gH
gH
gH
gpUL141
PAE (Å)
30
20
10
0

Fig. S4. AlphaFold3 (AF3) predictions of HCMV glycoprotein complexes. (A) Predicted structure of gH (blue semi-transparent surface) in complex with a homodimer of gpUL141 (orange semi-transparent surface). Proteins are shown as ribbons coloured by prediction confidence (predicted local distance difference test; pLDDT), from red (low confidence, pLDDT ≤ 50) to blue (high confidence, pLDDT ≥ 90). (B) Superposition of the predicted gpUL141:gH complex (light orange and blue, respectively) onto the experimental structure (PDB 9DIX[12]) of gpUL141 in complex with gH and gpUL116 (dark orange, blue and purple, respectively). For clarity, residues that were not modelled in the experimental structure are omitted from the AF3 model. The AF3 model is highly similar to the experimental structure (root mean squared deviation of 1.8 Å across 960 Cα residues). (C) Predicted complex between a homotrimer of gB (cyan semi-transparent surface) and a homodimer of gpUL141 (orange semi-transparent surface), with ribbons coloured as in (A). gB has been predicted in the post-fusion conformation[13]. Inset shows the predicted aligned error (PAE) of the complex, indicating low confidence in the orientation of gpUL141 relative to gB. (D) Predicted complex between a gH (blue semi-transparent surface) a UL116 (purple semi-transparent surface), with ribbons coloured as in (A). (E) Predicted structure of two gH molecules (blue semi-transparent surfaces) in complex with a homodimer of gpUL141 (orange semi-transparent surface). The PAE plot (inset) indicates that the relative orientations of gH molecules with respect to the gpUL141 homodimer are predicted with high confidence.

Table S1. Virus encoded proteins identified by MS-analysis of Merlin virions.

ORF[a]	Rank[b]	ID[c]	Protein/Function	AD169[e]	RhCMV[f]	MCMV[g]	TB40[h]	AD169-RV[i]
			**A- Proteins identified with high confidence**					
			Capsid:					
UL46	8	8/8	pUL46/mCBP (minor capsid protein binding protein); interacts with mCP to form capsid triplex	X	X	X	X	X
UL48A	17	8/8	pUL48A/SCP (smallest capsid protein); decorates capsid hexons	X	X	X	X	X
UL80	39	8/8	pUL80/PR-AP (assembly protein) precursor; scaffold for capsid assembly	X	X	X	X	X
UL85	6	8/8	pUL85/mCP (minor capsid protein); interacts with mCBP to form capsid triplex	X	X	X	X	X
UL86	3	8/8	pUL86/MCP (major capsid protein); forms capsid walls and faces	X	X	X	X	X
UL104	70	8/8	pUL104/PORT; forms specialized portal capsomere for genome insertion/release	X	X	X	X	X
			Tegument:					
UL23	67	6/8	Tegument. Inhibits transcription of IFN-γ stimulated genes					
UL24	19	8/8	pUL24; US22 family member; enhances replication in endothelial cells	X	X		X	X
UL25	7	8/8	pUL25; UL25 family member	X	X	X	X	X
UL26	16	8/8	pUL26; US22 family member, MIEP transcription transactivator, tegument material phosphorylation, stabilises virion	X	X			X
UL28/UL29	79	5/8	pUL28/UL29; US22 family member, Stimulates IE gene expression			X		X
UL32	12	8/8	pp150 / NSP (nucleocapsid-proximal stabilization protein); virion maturation	X	X	X	X	X
UL35	41	8/8	pUL35; UL25 family member, virion morphogenesis	X	X	X	X	X
UL36	50	7/8	pUL36x1; US22 family member, vICA (viral inhibitor of caspase-8-induced apoptosis)					
UL43		8/8	pUL43; US22 family member	X		X		X
UL45	15	8/8	pUL45/RR1 (Ribonucleotide reductase homologue sub-unit)	X	X	X	X	X
UL47	28	8/8	pUL47/LTPbp (Largest tegument protein binding protein); interacts with ppUL48 intra-cellular transport of nucleocapsid	X	X	X	X	X
UL48	33	8/8	pUL48/LTP (Largest tegument protein); interacts with ppUL47 intra-cellular transport of nucleocapsid	X	X	X	X	X

UL50	38	8/8	pUL50/NEC1 (nuclear egress complex membrane anchoring component 1); interacts with pUL53 (NEC2) to orchestrate nuclear egress of nucleocapsids	X	X			X
UL52	62	8/8	pUL52; DNA encapsidation, nucleocapsid formation		X			X
UL69	49	8/8	ppUL69; MRP (multiple regulatory protein)	X		X	X	X
UL71	47	8/8	pUL71; Secondary envelopment, virion egress	X	X	X	X	X
UL82	5	8/8	ppUL82/pp71/UMP (upper matrix protein); secondary envelopment, relieves DAXX-mediated repression	X	X	X	X	X
UL83	1	8/8	ppUL83/pp65/LMP (lower matrix protein); phosphorylase, innate and adaptive immune response evasion	X	X	X	X	X
UL88	34	8/8	pUL88; Putative cytoplasmic egress function	X	X	X	X	X
UL94	11	8/8	pUL94; interacts with pp28 to facilitate secondary envelopment	X	X	X	X	X
UL96	76	7/8	pUL96; interacts with pp150 to stabilise nucleocapsid during cytoplasmic egress	X	X			X
UL97	35	8/8	ppUL97/VPK (viral protein kinase); phosphorylates viral and cellular proteins	X	X	X	X	X
UL99	20	8/8	ppUL99/pp28; myristylated protein, secondary envelopment	X	X	X	X	X
UL103	54	7/8	pUL103; VEP (Virion and DB egress protein); orchestrates release from producer cells	X	X			X
IRS1	18	8/8	pIRS1; US22 family member, PKR (Protein kinase R) inhibitor, Transcriptional activator	X				X
US22	32	8/8	pUS22; US22 family member	X	X	X		X
US23	91	8/8	pUS23; US22 family member	X				X
US24	82	7/8	pUS24; US22 family member; enhances early infection	X	X			X
TRS1	85	8/8	pTRS1; US22 family member, PKR (Protein kinase R) inhibitor, Transcriptional activator, capsid assembly	X	X			X
	Transcription/replication machinery, yet to be assigned to virion compartment:							
UL34	57	8/8	pUL34; Represses US3 transcription					X
UL44	2	8/8	ppUL44/PPS (Viral DNA polymerase processivity sub-unit); DNA synthesis	X	X	X		X
UL57	55	8/8	ppUL57/SSB (single-stranded DNA binding protein); DNA synthesis	X		X		X
UL70	73	8/8	pUL70/HP2 (DNA helicase-primase sub-unit 2); DNA synthesis			X		X
UL77	72	8/8	pUL77/CVC1 (putative capsid vertex-specific component 1); DNA encapsidation	X	X	X		X
UL84	24	8/8	ppUL84/Viral DNA replication accessory; DURP family member, DNA synthesis	X	X			X
UL89	60	8/8	pUL89/TER1 (terminase sub-unit 1); DNA encapsidation	X			X	X
UL93	83	7/8	pUL93/CVC2 (putative capsid vertex-specific component 2); DNA encapsidation	X	X		X	X

UL98	44	8/8	pUL98/NUC (deoxyribonuclease)		X	X		X
UL112-UL113	40	8/8	pUL112/UL113; Orchestrates DNA synthesis, transcriptional activator	X	X			X
UL114	59	7/8	ppUL114; DNA synthesis UNG (Uracil-DNA glycosylase), control DNA synthesis					X
UL122	48	8/8	pUL122; IE2; transactivator or host-cell transcription machinery	X	X		X	X
UL146	66	7/8	gpUL146; vCXC chemokine homologue family, putative chemokine					
UL54	81	5/8	pUL54; POL; catalytic DNA polymerase sub-unit	X		X		X
UL56	86	6/8	pUL56; TER2 (terminase sub-unit 2); DNA encapsidation	X		X		X
UL102	87	5/8	pUL102/HP3 (helicase primase sub-unit 3); DNA synthesis		X	X		X
UL147	69	6/8	pUL147; vCXC-2 chemokine homologue family, putative chemokine					
Envelope:								
RL10	29	8/8	gpRL10	X			X	X
RL11	51	8/8	gpRL11/gp34; RL11 family member, IgG Fc binding					X
RL12	25	8/8	gpRL12/gp95; RL11 family member, IgG Fc binding					X
RL13	23	2/2	gpRL13; RL11 family member, putative IgG Fc binding, impedes replication in fibroblasts and epithelial cells		X			
UL16	30	7/8	gpUL16; membrane glycoprotein, NK cell evasion, blocks NKG2D ligands MICB, ULBP1 and ULBP2					
UL33x1	43	8/8	pUL33x1; GPCR homologue family member, constitutive signalling GPCR	X	X		X	X
UL37	53	5/8	Isoform vMIA of pUL37; multifunctional transmembrane protein that plays several key roles in viral replication					
UL41A	31	7/8		X	X		X	X
UL55	13	8/8	gB; gCI subunit, virion binding and entry	X	X	X	X	X
UL73	21	8/8	gN; gCII sub-unit, virion binding, progeny virion secondary envelopment	X	X			X
UL74	92	8/8	gO; gCIII sub-unit, fibroblast cell entry, release of progeny virions from producer cells	X	X	X		X
UL74A	58	6/8	gpUL74A; putative membrane glycoprotein					X
UL75	37	8/8	gH; gCIII/pentameric complex sub-unit, entry into cells	X	X	X		X
UL78	74	8/8	gpUL78; GPCR homologue family member; putative chemokine receptor					
UL100	87	8/8	gM; gCII sub-unit, virion binding, progeny virion secondary envelopment	X	X	X	X	X
UL115	52	7/8	gL; gCIII/pentameric complex sub-unit, entry into cells	X	X			X

UL116	42	8/8	gpUL116; putative membrane glycoprotein		X			X
UL118-UL119	78	8/8	gpUL118-UL119/gp68; IgG Fc binding	X	X		X	X
UL128	45	5/6	gpUL128; pentameric complex, entry into non-fibroblasts				X	X
UL130	63	5/6	gpUL130; pentameric complex, entry into non-fibroblasts					
UL131A	75	6/6	gpUL131A; pentameric complex, entry into non-fibroblasts				X	
UL132	10	8/8	gpUL132; enhances replication in fibroblasts	X	X		X	X
UL139	94	5/8	gpUL139; putative membrane glycoprotein					
UL140	95	6/8	pUL140; putative membrane protein					
UL141	9	7/7	gpUL141; UL14 family member, NK evasion, down regulates CD155 and CD112		X			
UL148	14	7/8	gpUL148, putative membrane glycoprotein					
US9	46	7/8	gpUS9; US6 family member, membrane glycoprotein					
US12	65	8/8	pUS12; US12 family member, 7TM protein		X			X
US13	77	6/8	pUS13; US12 family member, 7TM protein					
US14	80	8/8	pUS14; US12 family member, 7TM protein					X
US20	27	8/8	pUS20; US12 family member, 7TM protein					X
US27	26	8/8	gpUS27; GPCR homologue family member, 7TM protein, enhances virion release	X			X	
US28	56	8/8	gpUS28; GPCR homologue family member, 7TM protein, CC and CXC3 chemokine receptor		X			X
Uncharacterised:								
RL1	68	5/8	pRL1; RL1 family member, Degrades the host antiviral factor SLFN11 via the cullin4-RING E3 ubiquitin ligase (CRL4) complex					
UL13	88	6/8	pUL13; modulates mitochondrial ultrastructure		X			
UL31	90	7/8	pUL31; DURP family member			X		
UL95	89	7/8	Participates in the expression of late viral mRNAs			X		
UL135	97	5/8	pUL135; reactivation from latency, actin remodelling					
UL145	22	7/8	pUL145; recruit DDB1-containing ubiquitin ligases to induce proteasomal degradation of STAT2.					
UL148D	36	6/8	Inhibits ADAM17 function				X	
UL150A	71	6/8	Putative secreted protein					

US1	84	7/8	US1 family member		X		X
US8	61	7/8	gpUS8; Type I membrane glycoprotein				
US26	93	6/8	pUS26; US22 family member		X		
**B- Proteins identified with low confidence**							
Transcription/replication machinery, immune evasions, yet assigned to virion compartment:							
UL105		4/8	pUL105; HP1 (helicase-primase sub-unit 1), DNA synthesis			X	
UL123		4/8	ppUL123/IE1 (major IE protein); promotes transcription cascade, enhances IE2 activation				X
Envelope:							
UL15A		1/8	Uncharacterised				
UL40		2/8	Loads peptides onto HLA-E				
US3		4/8	Retains MHC class I heterodimers in the endoplasmic reticulum				
US18		4/8	pUS18; US12 family member, 7TM protein		X		
Uncharacterised							
RL9A		1/8					
UL5		2/8	May play a role in rearrangement of cellular cytoskeleton towards an efficient viral assembly and spreading.	X			
UL14		4/8	Uncharacterised				
UL17		3/8					
UL22A		3/8	Glycoprotein	X			X
UL27		1/8	Uncharacterised				
UL30		1/8	Uncharacterised		X		
UL49		4/8	pUL49; subunit of the viral pre-initiation complex, regulates gene transcription	X			
UL53		4/8	pUL53 (NEC2) (nuclear egress complex membrane anchoring component 2); interacts with pUL50/NEC1 to orchestrate nuclear egress of nucleocapsids				X
UL72		2/8	Deoxyuridine 5-triphosphate nucleotidohydrolase	X		X	
UL76		4/8	pUL76; modulates gene expression				
UL117		1/8	Plays a role in the inhibition of host DNA replication in the infected cell				X
UL136		1/8	Plays a role in latency				

UL138	3/8	Modulates the expression of several host cell surface receptors
UL150	1/8	Uncharacterised
US30	1/8	Uncharacterised
US32	4/8	Uncharacterised

[a] ORFs encoding proteins identified in Merlin virions.
[b] rank of protein based on abundance in the wildtype HCMV virion proteome, with 1 being the most abundant as assessed by IBAQ.
[c] identities depict the number of virion preparations in which proteins were detected.
[d] comments as to the biochemistry and function of proteins identified, as described in Field's Virology
[e] proteins previously identified during mass spectrometry analysis of HCMV Strain AD169 virions[14]
[f] homologous proteins identified during mass spectrometry analysis of RhCMV virions[15]
[g] homologous proteins identified during mass spectrometry analysis of MCMV virions[16]
[h] homologous proteins identified during mass spectrometry analysis of HCMV Strain TB40 virions[17]
[i] proteins previously identified during mass spectrometry analysis of HCMV Strain AD169 virions[18]

Dataset S1 (separate file). Mass spectrometry analysis of Merlin virions.

Dataset S2 (separate file). (A) HCMV proteins detected in virion or WCL preparations, or both. (B) Comparison of abundance of human proteins detected in virion and WCL preparations. (C) Significantly enriched clusters and their components from DAVID analysis of human proteins enriched with a virion : WCL ratio of >2.

Dataset S3 (separate file). Mass spectrometry analysis of SILAC IP of gpUL141 from Merlin Virions.

SI References

1. Tischer, B.K., von Einem, J., Kaufer, B., and Osterrieder, N. (2006). Two-step red-mediated recombination for versatile high-efficiency markerless DNA manipulation in Escherichia coli. Biotechniques *40*, 191-197. 10.2144/000112096.
2. Stanton, R.J., Baluchova, K., Dargan, D.J., Cunningham, C., Sheehy, O., Seirafian, S., McSharry, B.P., Neale, M.L., Davies, J.A., Tomasec, P., et al. (2010). Reconstruction of the complete human cytomegalovirus genome in a BAC reveals RL13 to be a potent inhibitor of replication. The Journal of clinical investigation *120*, 3191-3208. 10.1172/JCI42955.
3. Murrell, I., Wilkie, G.S., Davison, A.J., Statkute, E., Fielding, C.A., Tomasec, P., Wilkinson, G.W., and Stanton, R.J. (2016). Genetic Stability of Bacterial Artificial Chromosome-Derived Human Cytomegalovirus during Culture In Vitro. J Virol *90*, 3929-3943. 10.1128/JVI.02858-15.
4. Kerr-Jones, L.E., Soday, L., Hughes, N.C., Wang, X., Hunter, L.H., Antrobus, R., Miners, K.L., Ladell, K., Price, D.A., Fielding, C.A., et al. (2024). Highly Multiplexed Proteomic Analysis of HCMV-Infected Dendritic Cells Reveals Global Manipulation of Adaptive Immunity and Host Restriction of Viral Replication. bioRxiv, 2024.2004.2030.591855. 10.1101/2024.04.30.591855.
5. Abramson, J., Adler, J., Dunger, J., Evans, R., Green, T., Pritzel, A., Ronneberger, O., Willmore, L., Ballard, A.J., Bambrick, J., et al. (2024). Accurate structure prediction of biomolecular interactions with AlphaFold 3. Nature *630*, 493-500. 10.1038/s41586-024-07487-w.
6. Emsley, P., Lohkamp, B., Scott, W.G., and Cowtan, K. (2010). Features and development of Coot. Acta Crystallographica Section D *66*, 486-501. doi:10.1107/S0907444910007493.
7. Tomasec, P., Wang, E.C., Davison, A.J., Vojtesek, B., Armstrong, M., Griffin, C., McSharry, B.P., Morris, R.J., Llewellyn-Lacey, S., Rickards, C., et al. (2005). Downregulation of natural killer cell-activating ligand CD155 by human cytomegalovirus UL141. Nature immunology *6*, 181-188. 10.1038/ni1156.
8. Zhou, M., Yu, Q., Wechsler, A., and Ryckman, B.J. (2013). Comparative analysis of gO isoforms reveals that strains of human cytomegalovirus differ in the ratio of gH/gL/gO and gH/gL/UL128-131 in the virion envelope. J Virol *87*, 9680-9690. 10.1128/JVI.01167-13.
9. Laib Sampaio, K., Stegmann, C., Brizic, I., Adler, B., Stanton, R.J., and Sinzger, C. (2016). The contribution of pUL74 to growth of human cytomegalovirus is masked in the presence of RL13 and UL128 expression. The Journal of general virology *97*, 1917-1927. 10.1099/jgv.0.000475.

10. Calo, S., Cortese, M., Ciferri, C., Bruno, L., Gerrein, R., Benucci, B., Monda, G., Gentile, M., Kessler, T., Uematsu, Y., et al. (2016). The Human Cytomegalovirus UL116 Gene Encodes an Envelope Glycoprotein Forming a Complex with gH Independently from gL. J Virol *90*, 4926-4938. 10.1128/JVI.02517-15.
11. Gerna, G., Sarasini, A., Patrone, M., Percivalle, E., Fiorina, L., Campanini, G., Gallina, A., Baldanti, F., and Revello, M.G. (2008). Human cytomegalovirus serum neutralizing antibodies block virus infection of endothelial/epithelial cells, but not fibroblasts, early during primary infection. The Journal of general virology *89*, 853-865. 10.1099/vir.0.83523-0.
12. Norris, M.J., Henderson, L.A., Siddiquey, M.N.A., Yin, J., Yoo, K., Brunel, S., Mor, M., Saphire, E.O., Benedict, C.A., and Kamil, J.P. (2025). The GATE glycoprotein complex enhances human cytomegalovirus entry in endothelial cells. Nat Microbiol. 10.1038/s41564-025-02025-4.
13. Chandramouli, S., Ciferri, C., Nikitin, P.A., Calo, S., Gerrein, R., Balabanis, K., Monroe, J., Hebner, C., Lilja, A.E., Settembre, E.C., and Carfi, A. (2015). Structure of HCMV glycoprotein B in the postfusion conformation bound to a neutralizing human antibody. Nature communications *6*, 8176. 10.1038/ncomms9176.
14. Varnum, S.M., Streblow, D.N., Monroe, M.E., Smith, P., Auberry, K.J., Pasa-Tolic, L., Wang, D., Camp, D.G., 2nd, Rodland, K., Wiley, S., et al. (2004). Identification of proteins in human cytomegalovirus (HCMV) particles: the HCMV proteome. J Virol *78*, 10960-10966. 10.1128/JVI.78.20.10960-10966.2004.
15. Malouli, D., Nakayasu, E.S., Viswanathan, K., Camp, D.G., 2nd, Chang, W.L., Barry, P.A., Smith, R.D., and Fruh, K. (2012). Reevaluation of the coding potential and proteomic analysis of the BAC-derived rhesus cytomegalovirus strain 68-1. J Virol *86*, 8959-8973. 10.1128/JVI.01132-12.
16. Kattenhorn, L.M., Mills, R., Wagner, M., Lomsadze, A., Makeev, V., Borodovsky, M., Ploegh, H.L., and Kessler, B.M. (2004). Identification of proteins associated with murine cytomegalovirus virions. J Virol *78*, 11187-11197. 10.1128/JVI.78.20.11187-11197.2004.
17. Bogdanow, B., Gruska, I., Mühlberg, L., Protze, J., Hohensee, S., Vetter, B., Bosse, J.B., Lehmann, M., Sadeghi, M., Wiebusch, L., and Liu, F. (2023). Spatially resolved protein map of intact human cytomegalovirus virions. Nature Microbiology *8*, 1732-1747. 10.1038/s41564-023-01433-8.
18. Coute, Y., Kraut, A., Zimmermann, C., Buscher, N., Hesse, A.M., Bruley, C., De Andrea, M., Wangen, C., Hahn, F., Marschall, M., and Plachter, B. (2020). Mass Spectrometry-Based Characterization of the Virion Proteome, Phosphoproteome, and Associated Kinase Activity of Human Cytomegalovirus. Microorganisms *8*. 10.3390/microorganisms8060820.
